# Buoyancy and hydrostatic balance in a West Indian Ocean coelacanth *Latimeria chalumnae*

**DOI:** 10.1186/s12915-022-01354-8

**Published:** 2022-08-19

**Authors:** Henrik Lauridsen, Jens Mikkel Hyllested Pedersen, Steffen Ringgaard, Peter Rask Møller

**Affiliations:** 1 Department of Clinical Medicine, Aarhus University, Palle Juul-Jensens Boulevard 99, 8200 Aarhus N, Denmark; 2 Natural History Museum of Denmark, University of Copenhagen, Copenhagen, Denmark; 3 Norwegian College of Fishery Science, UiT - The Arctic University of Norway, Tromsø, Norway

**Keywords:** Ecophysiology, Depth regulation, Bone mineral density, Lipid accumulation, Fatty organ, Headstand, Magnetic resonance imaging, Computed tomography, Magnetic resonance spectroscopy

## Abstract

**Background:**

Buoyancy and balance are important parameters for slow-moving, low-metabolic, aquatic organisms. The extant coelacanths have among the lowest metabolic rates of any living vertebrate and can afford little energy to keep station. Previous observations on living coelacanths support the hypothesis that the coelacanth is neutrally buoyant and in close-to-perfect hydrostatic balance. However, precise measurements of buoyancy and balance at different depths have never been made.

**Results:**

Here we show, using non-invasive imaging, that buoyancy of the coelacanth closely matches its depth distribution. We found that the lipid-filled fatty organ is well suited to support neutral buoyancy, and due to a close-to-perfect hydrostatic balance, simple maneuvers of fins can cause a considerable shift in torque around the pitch axis allowing the coelacanth to assume different body orientations with little physical effort.

**Conclusions:**

Our results demonstrate a close match between tissue composition, depth range and behavior, and our collection-based approach could be used to predict depth range of less well-studied coelacanth life stages as well as of deep sea fishes in general.

**Supplementary Information:**

The online version contains supplementary material available at 10.1186/s12915-022-01354-8.

## Background

Coelacanths represent a group of lobe-finned fishes (sarcopterygians) dating back to the early Devonian (410 Myr) with two known extant species, *Latimeria chalumnae* Smith, 1939 and *L. menadoensis* Pouyaud, Wirjoatmodjo, Rachmatika, Tjakrawidjaja, Hadiaty & Hadie, 1999 [1]. Fossilized remains of extinct coelacanths have been found in a range of freshwater, brackish, and marine environments [2], but extant species are found exclusively in moderately deep marine waters [3–6]. The first discovery of a living coelacanth in South Africa in 1938 [7] and rediscovery in 1952 in the Comoros [8] sparked a great interest in the anatomy and physiology of this Lazarus taxon culminating in several research papers and a major anatomical atlas in three volumes based on thorough dissections and X-ray radiographs [9–11]. The first reported use of non-invasive imaging techniques, computed X-ray tomography (CT), and magnetic resonance imaging (MRI) in fishes was on a coelacanth specimen (Coelacanth Conservation Council, CCC 141) [12–15]. Since then, the external and internal anatomy of an ever increasing number of both fresh and preserved fish species have been digitized in these ways and more coelacanth specimens have undergone careful CT and MRI examinations [16–18]. As Hilton and others have observed, the adoption of imaging technologies, originally developed for medical use, by ichthyologists to reveal internal anatomy of fishes in some way parallels the clearing and staining revolution of the 1960s and 1970s [15, 19]. However, CT and MRI are fundamentally different from single projection-based imaging approaches such as photography of cleared specimens or simple X-ray images, in that these technologies, if used carefully, provide the potential to generate quantitative measurements on tissue components [20]. Compared to the large amount of strictly morphology data generated on fish and other vertebrates using these imaging techniques, this fact seems highly underappreciated.

Buoyancy and hydrostatic balance are important parameters for all species of aquatic macrofauna. A negatively or positively buoyant organism will continuously need to expend energy to maintain vertical position in the water column, and only a neutrally buoyant organism can effortlessly keep station. In the same way, in a hydrostatically unstable organism, an external force will result in the organism falling out of its original orientation and needing to expend energy to maintain a particular, fixed orientation. In contrast, a hydrostatically stable organism will, by virtue of its geometry and distribution of mass, automatically generate forces to counteract a perturbing force and in this way return it to its original orientation [21]. While several observations on both species of known extant coelacanths indicate neutral or close to neutral buoyancy and hydrostatic stability in *Latimeria* (Additional file 1) [22–35], this has to our knowledge never been tested. A simple way to physically measure buoyancy of an object relative to a surrounding medium, e.g., a fish in water, is to weigh the object in and out of the medium. The relative buoyancy can be expressed as the object’s weight in the medium divided by its weight out of the medium [36]. A neutrally buoyant object has a relative buoyancy of 0%. Hydrostatic stability can be physically measured by determining the relative positions of the center of gravity to the center of buoyancy using the plumb line method with the object in and out of the medium [21, 37]. To the best of our knowledge, these procedures have never intentionally been carried out on a freshly caught coelacanth. But even if they had, they would only provide limited information on buoyancy and hydrostatic balance of the coelacanth in its natural environment some hundred meters below the tropical water surface [3–6]. As temperature drops and pressure increases at increasing depths, density properties of the main tissue components—bone, lean tissue (muscles, cartilage, connective tissue, etc.), and fatty tissue—vary differently [38, 39]. Bone is generally considered incompressible and whereas the density change of watery lean tissues parallels that of the surrounding sea water, density of body lipids is both more sensitive to temperature and pressure changes [39]. In addition to temperature and pressure effects on the density of sea water providing buoyancy, changes in salinity at varying depths also affects density of displaced water and thus buoyancy forces. To directly *measure* buoyancy and hydrostatic balance in the coelacanths in its natural environment, one would need to conduct the procedures described above at depth, which is currently unachievable. An alternative route to *estimate* these two parameters is by that of modeling. When content and distribution of the main body tissues are known at a sufficient level of detail, e.g., by the use of quantitative CT to image mineralized structures and quantitative MRI to image water- and lipid-rich structures, it is possible to calculate buoyancy and hydrostatic balance at different depths (different pressures) when the physical parameters of temperature and salinity are available at these depths. Additionally, by digitally segmenting the coelacanth body into moveable objects (pectoral, pelvic, dorsal, anal, and caudal fins) and reputedly buoyancy-specific structures, such as the fatty organ, it is possible to model how different body postures affect hydrostatic balance and the role of potential anatomical adaptations to obtain neutral buoyancy.

## Results

### Bone mineral and lipid content measurements on preserved specimens

Before attempting to extract absolute measurements of bone mineral and lipid contents of the preserved coelacanth specimen using non-invasive MRI and CT imaging, we first needed to establish any potential effects that preservation may have on image acquisition as well as on actual bone mineral and lipid content. We found preservation to have negligible effect on actual bone mineral and lipid content of preserved specimens, but CT and MRI-based measurements of both bone mineral and lipid content needed to be corrected for the effect of ethanol storage (Additional files 2, 3 and 4).

### Composition and density of tissues in the coelacanth

**Fig. 1:**
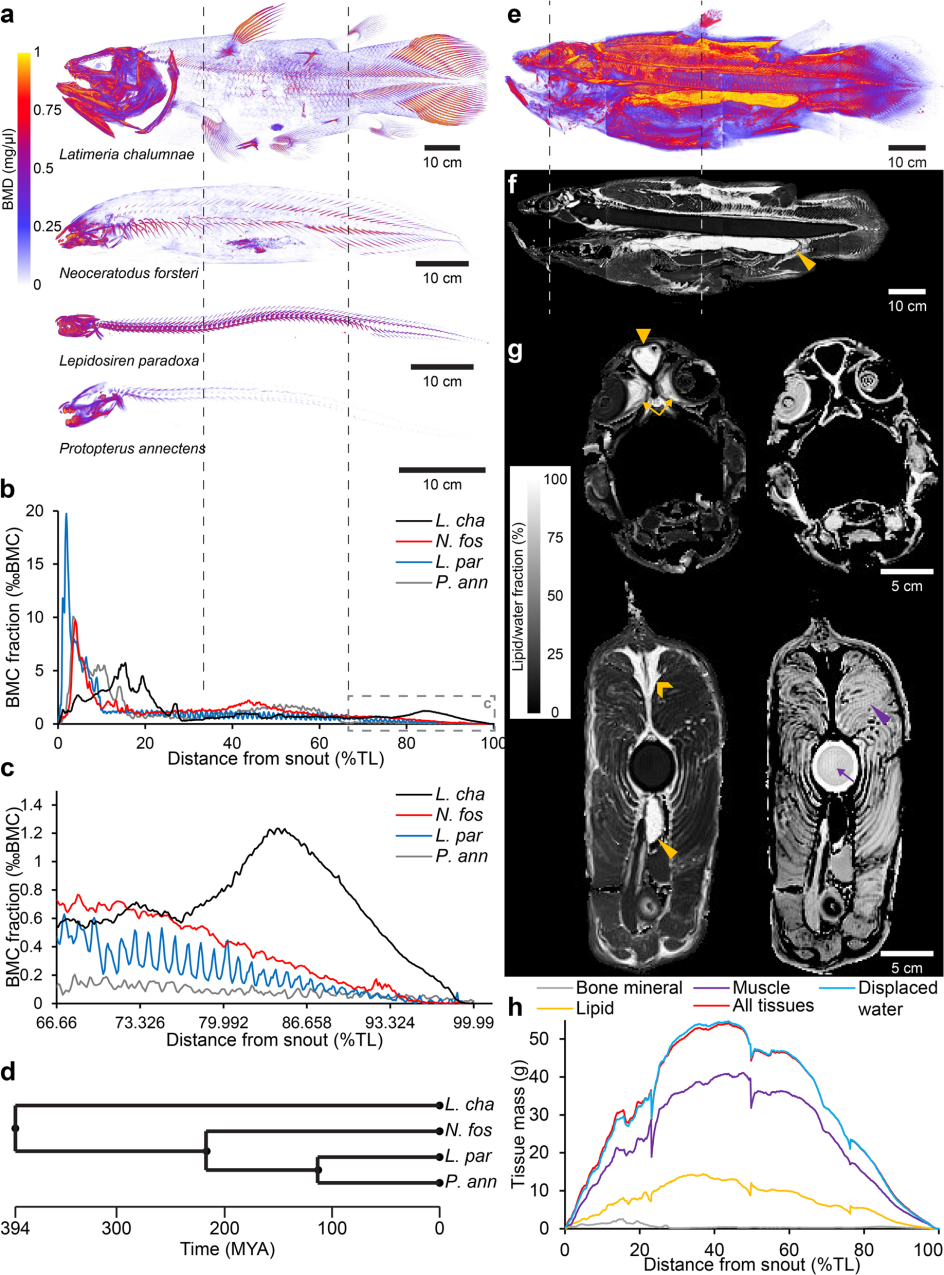
Distribution of bone mineral, lipid and muscle tissue in the coelacanth. **a** Volume rendering of a three-dimensional bone mineral density (BMD) map of the coelacanth and three extant species of lungfishes. **b,c** Distribution of bone mineral content (BMC) fraction of total BMC along the total length (TL) in the coelacanth and lungfishes. The dashed gray square in **b** is magnified in **c**. **d** Putative time calibrated phylogeny of extant non-tetrapod sarcopterygians. **e** Volume rendering of a three-dimensional lipid fraction map of the coelacanth. Same color scale as in **a** is used for lipid fraction ranging from 0 to 100%. **f,g** Sagittal (**f**) and transversal (**g**) sections of lipid fraction (**f** and leftmost sections in **g**) and water fraction (rightmost sections in **g**). Yellow isosceles triangles point to the lipid-filled fatty organ, yellow equilateral triangle points to the lipid-filled pericerebral space, yellow arrows point to the lipid-filled post ocular space, yellow arrow head points to the lipid-filled perivertebral space, purple isosceles triangle points to the water-rich muscle, purple arrow points to the water-rich notochord. **h** Distribution of mass of different tissue types along the total length in the coelacanth. Here, all water-rich lean tissues are included in “muscle”

Mineralization of the coelacanth skeleton was mapped using quantitative CT. The coelacanth skeleton is unusual among extant non-tetrapod sarcopterygians in that it mainly exhibits mineralization at the extreme ends of the craniocaudal axis (Fig. 1a–d) (see also Meunier and colleagues [40]). This characteristic is shared to some degree in unrelated actinopterygians such as the notothenioid *Dissostichus eleginoides* and the trachichthyiform *Hoplostethus atlanticus* (Additional files 5 and 6). In the coelacanth, a heavily mineralized head region is separated from a similarly although somewhat less heavily mineralized caudal region by a mid-portion containing very little bone mineral with the only exception of the somewhat mineralized skeleton of the 1st dorsal fin (Fig. 1a; Additional file 7). The skeleton of the paired fins and the 2nd dorsal and anal fins contain very little bone mineral (Fig. 1a; Table 1; Additional file 7).

**Table 1:** Absolute (in kg) and relative (in %) content of bone mineral, lipid tissue, and lean tissue in different appendages and organs

Structure	All tissues (kg)	Bone mineral (kg)	Lipid tissue (kg)	Lean tissue (kg)	Displaced seawater (kg)	Bone mineral frac. (%)	Lipid frac. (%)	Lean frac. (%)
Complete body	26.02	0.45	6.13	19.45	26.12	1.72	23.54	74.74
	**(%)**	**(%)**	**(%)**	**(%)**	**(%)**	**(%)**	**(%)**	**(%)**
Main body	80.54	76.78	80.55	80.63	80.61	1.64	23.54	74.82
Fatty organ	0.63	0	2.51	0.06	0.74	0.00	93.46	6.54
Pectoral fins	0.78	0.91	0.56	0.84	0.76	2.02	16.69	81.29
Pelvic fins	0.80	0.49	0.68	0.85	0.80	1.05	19.91	79.04
1st dorsal fin	0.27	2.76	0.23	0.23	0.23	17.38	19.47	63.16
2nd dorsal fin	0.61	0.52	0.31	0.70	0.59	1.46	12.08	86.46
Anal fin	0.78	0.51	0.50	0.88	0.77	1.11	15.08	83.81
Caudal fin combined	14.00	16.34	13.43	14.12	13.93	2.00	22.58	75.41
Ventral muscle	–	–	–	–	–	–	12.77	87.23
Dorsal muscle	–	–	–	–	–	–	10.15	89.85
Intermuscular fat	–	–	–	–	–	–	68.13	31.87
Fatty organ, anterior	–	–	–	–	–	–	94.67	5.33
Fatty organ, posterior	–	–	–	–	–	–	96.67	3.33
Pericardial	–	–	–	–	–	–	83.68	16.32
Pericerebral	–	–	–	–	–	–	89.76	10.24
Perivertebral	–	–	–	–	–	–	89.70	10.30
Postocular	–	–	–	–	–	–	84.03	15.97
Liver	–	–	–	–	–	–	14.48	85.52
Spleen	–	–	–	–	–	–	13.48	86.52
Notochord, medulla	–	–	–	–	–	–	9.52	90.48
Notochord, cortex	–	–	–	–	–	–	2.36	97.64
Vestigial lung	–	13.2 mg	–	–	–	0.0030	5.83	94.17

Percentages in columns 2–6 refers to percentage out of complete body mass of the given tissue type, whereas percentages in columns 7–9 refers to percentages of the given tissue types within the appendage or organ, e.g., in the 1st dorsal fin, bone mineral constitutes 17.38% of the total tissue mass of the appendage which is 2.76% of the entire bone mineral in the specimen. Measurements on body segments (main body to caudal fin combined) are based on all voxels within the segment, whereas measurements on organs (ventral muscle to vestigial lung) are based on regions of interest within the specific organs. The exception is the bone mineral content of the vestigial lung (13.2 mg) which is the entire bone mineral content of that organ. Abbreviations: *fract.* fraction

Lipids are deposited in the coelacanth body as both large well-distinguished structures such as postocular, pericerebral, and perivertebral deposits and notably the large lipid-filled fatty organ (Fig. 1e–h; Table 1; Additional file 8), as well as intermuscular sheets of lipid-rich tissue most likely within the connective tissue of the myosepta (Fig. 1g, bottom; Table 1; Additional file 8). The cellular status of lipid deposits (i.e., intra- or extracellular) could not be observed with the non-invasive imaging methods. In addition to the non-invasive measurements, intramuscular lipid content was measured using lipid extraction on a ventral muscle sample and like non-invasive measurements this revealed a low lipid content of muscles (4.34% of wet weight and 19.55% of dry weight).

### Buoyancy at varying depths

**Fig. 2:**
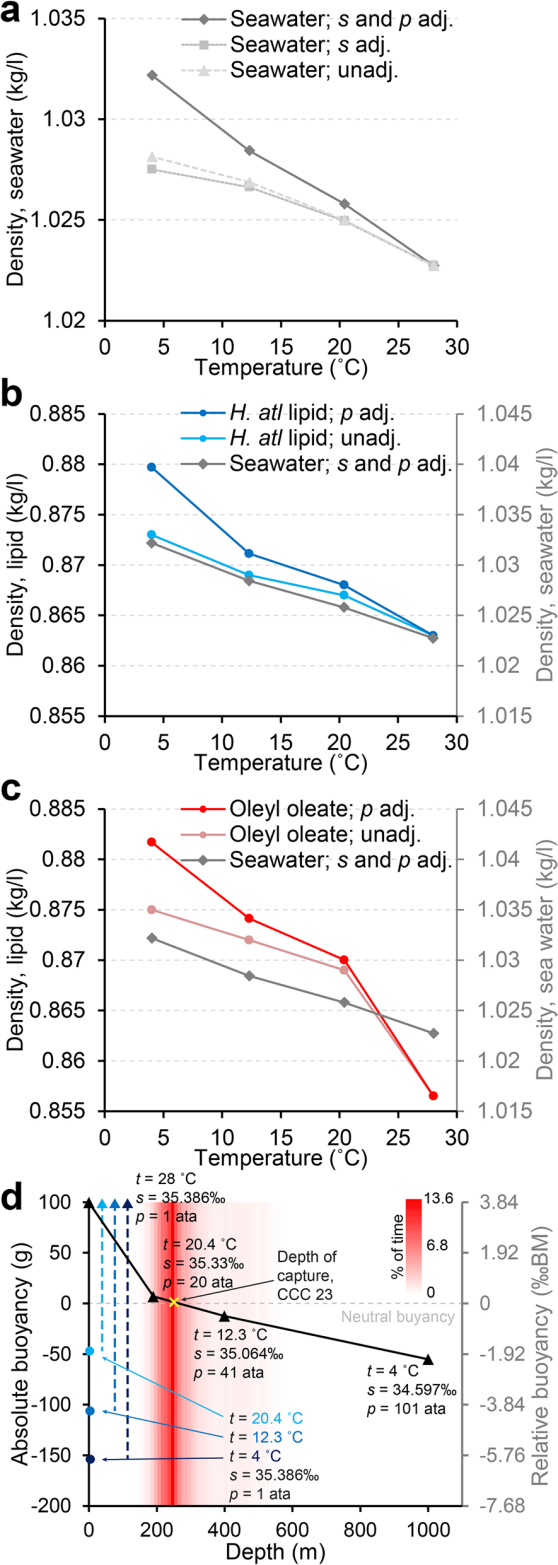
Effect of temperature, salinity, and pressure on density of seawater and lipids and on buoyancy of the coelacanth. **a** Density of Comoran seawater as a function of temperature with and without salinity (*s*) and pressure (*p*) adjustments (adj.). **b** Density of swim bladder lipid of *Hoplostethus atlanticus* (left vertical axis) as a function of temperature with and without pressure adjustment compared to density of Comoran seawater (right vertical axis). Notice that the density of the swim bladder lipid is more sensitive to pressure than seawater. Both vertical axes span 0.03 kg/l. **c** Density of oleyl oleate, the most prevalent wax ester in coelacanth (left vertical axis) as a function of temperature with and without pressure adjustment compared to density of Comoran seawater (right vertical axis). Notice that the density of oleyl oleate is more sensitive to pressure and temperature than seawater. Both vertical axes span 0.03 kg/l. **d** Absolute (left vertical axis) and relative (right vertical axis) buoyancy of the coelacanth at varying depths in Comoran waters. Prevalence (% of time) of coelacanths at different depths previously determined by ultrasonic telemetry [4] is presented in shades of red in the background of the graph. In its natural habitat at 190–400 m of depth, the coelacanth is close to neutrally buoyant. Venturing deeper, the coelacanth will become negatively buoyant. If brought rapidly to the surface (e.g., line fishing), the relatively cold coelacanth will initially be negatively buoyant but gradually become positively buoyant as it heats up to surface water temperature (exemplified from 190, 400, and 1000 m of depth to surface with increasingly dark blue circles and dashed lines)

As in any other living organism, the density of coelacanth tissues varies with temperature and ambient pressure. Similarly, the density of seawater varies with temperature, pressure, and salinity. Thus, the overall buoyancy of an aquatic organism at any given depth will be determined by the difference between the density of the overall body of the organism and the density of the displaced water. Using pure oleyl oleate (the dominant wax ester found in coelacanth tissue [34]) and extracted swim bladder lipid of *Hoplostethus atlanticus* (dominated by a mixture of wax esters quite similar to those of the coelacanth [41]) as proxies for overall lipids of the coelacanth, we measured density changes in these lipids relative to Comoran sea water in the range of temperatures and pressures that the coelacanth encounters in its normal vertical range of its habitat 190–400 m below sea level [42] as well as in extreme events like rapid surfacing and a 1000-m dive. Densities of both lipids were more sensitive to temperature and pressure changes than seawater (Fig. 2a–c). In subsequent analyses, the measured density of oleyl oleate was used as the best proxy for lipid density changes during vertical displacement of the coelacanth. At sea level (1 atmospheres absolute, ata) and water temperatures typically found at the surface of the most well described habitat of the coelacanth at the Comoro Islands (28 °C) [42], a temperature equilibrated coelacanth is positively buoyant with a relative buoyancy to body mass of + 3.83‰ (Fig. 2d). At the depth that the vast majority of coelacanths have been observed and fished, 190–400 m [42, 43], the coelacanth is close to neutrally buoyant, with a relative buoyancy ranging from + 0.26 to − 0.49‰ (Fig. 2d). Coelacanths have been tracked to a maximum depth of 698 m using ultrasonic telemetry [4], which fits well with the maximum depth of captures [43], although the latter is likely biased by limitations in fishing equipment. However, given the difficulties in deep sea observations, it may be that the coelacanth occasionally dives deeper. During an extreme dive of 1000 m, a temperature equilibrated coelacanth would be predicted to be negatively buoyant with a relative buoyancy of − 2.12‰ (Fig. 2d).

The abovementioned values of relative buoyancy rely on a coelacanth that is temperature equilibrated with the ambient seawater. Whereas the compacting force of pressure on soft tissues and its effect on tissue density is instantaneously alleviated if the coelacanth is moved between depths, a large-bodied organism such as the adult coelacanth will only slowly equilibrate to ambient temperature. This results in cold and denser soft tissue in warm and less dense seawater if a coelacanth is lifted rapidly from its typical depth of habitat to the surface, e.g., if captured on a fishing line. A coelacanth lifted from depths of 190, 400, or 1000 m to the surface will initially be negatively buoyant, relative buoyancy of − 1.81‰ (190 m), − 4.08‰ (400 m), − 5.92‰ (1000 m), but over time as the temperature of the coelacanth tissue is equilibrated to ambient temperature, it will become positively buoyant (+ 3.83‰) (dashed lines in blue nuances in Fig. 2d).

### Hydrostatic balance at different depths and body postures

**Fig. 3:**
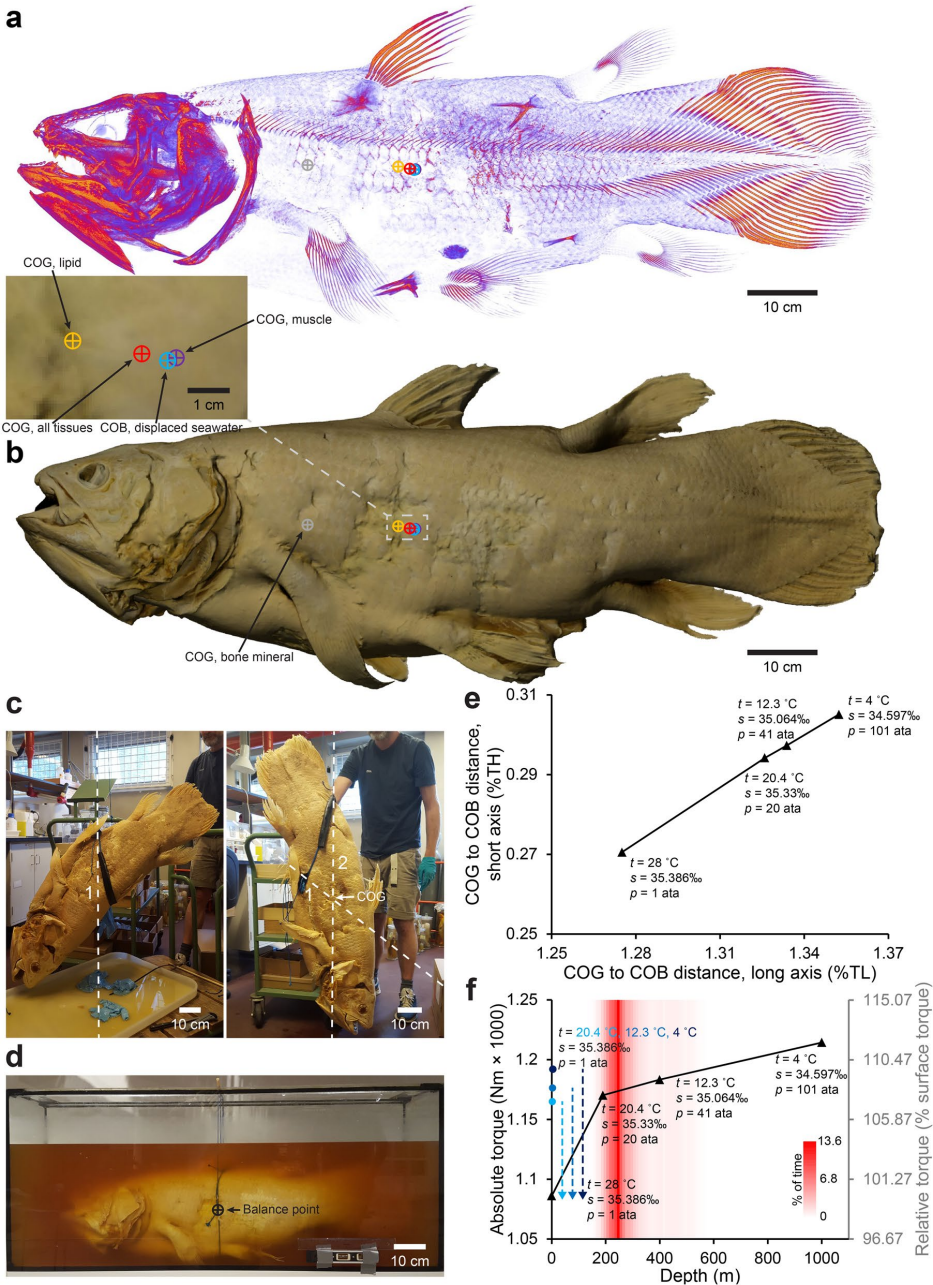
Hydrostatic balance of the coelacanth at different depths. **a,b** Modeled overall center of gravity (COG) (red reticle), overall center of buoyancy (COB) (blue reticle), bone mineral COG (gray reticle), lipid COG (yellow reticle), and muscle COG (purple reticle) overlaid on bone mineral volume reconstruction (**a**) and photogrammetry reconstructed surface model (**b**) of the coelacanth. **c** Physical measurement of COG using the plumb line method at surface pressure and room temperature of 70% v/v EtOH preserved coelacanth. The intersection of the extrapolated vertical lines from two different anchor points (left photo: ventral side, caudal to the pelvic fins; right photo: dorsal side, caudal to the 2nd dorsal fin) reveal the COG (under the assumption of a negligible buoyancy force provided by the displaced atmospheric air). **d** Balance point of coelacanth immersed in its 70% EtOH preservation fluid. A line that was adjustable in the long axis of the specimen was tied around the circumference of the specimen and the specimen was hung from two vertical lines with adjustable connections to the circumferential line in the dorsoventral (short) axis. Long and short axis position of the anchor points were adjusted until the specimen was in balance indicating the balance point for the specimen in the preservation fluid between the COG and COB. **e** Distances between COG and COB relative to total length (TL) and total height (TH) in the long and short axis at different depths with varying temperatures, pressures, and salinities. **f** Absolute and relative torque (relative to torque at surface) at different depths with varying temperatures, pressures, and salinities. Distance between the COG and COB in both long and short axis increases slightly with depth in the modeled depth range resulting in increased torque and the need to produce extra hydrodynamic force to counteract the hydrostatic force acting to push COB directly above COG. If brought rapidly to the surface, the GOG to GOB distance will gradually decrease as the coelacanth heats up to surface temperatures resulting in a decreased torque (exemplified from 190, 400, and 1000 m of depth to surface with increasingly dark blue circles and dashed lines)

The centers of gravity of the hard and soft tissue compartments of the coelacanth are located at different positions (Fig. 3a, b; Additional file 9). Due to the heavily calcified skull, the center of gravity of bone mineral is positioned in the anterior portion of the coelacanth body 33.61% of the total length from the snout and 49.58% of the total height from the most dorsal portion position (gray reticle in Fig. 3a, b). Centers of gravity of lipids and water-rich lean tissues are positioned at 44.04% and 46.04%, respectively, of the total length from the snout and 49.95% and 50.84% of the total height from the most dorsal position (yellow and purple reticles in Fig. 3a, b). In total, this yields a modeled center of gravity of the coelacanth body at 45.36% of the total length from the snout and 50.61% of the total height from the most dorsal position (red reticle in Fig. 3a, b). This is closely matched by a physical measurement of center of gravity and balance point at surface pressure and room temperature (Fig. 3c, d; Additional file 10). Center of gravity of displaced seawater which equals the center of buoyancy of the coelacanth is in close proximity to its center of gravity (blue reticle in Fig. 3a, b; Additional file 9), but varies with depth as lipid-rich tissues compress more than water-rich lean tissue and seawater [39]. Thus, the distance between center of buoyancy and center of gravity increases at increasing depths (Fig. 3e; Additional file 9). At surface pressure and temperature, center of buoyancy is positioned 1.28‰ of the total length caudal to and 0.27‰ of total height dorsal to the center of gravity. This results in a modest torque of 1.09 Nm × 1000 in a horizontally oriented coelacanth which gradually increases to 1.21 Nm × 1000 at 1000 m of depth (Fig. 3f; Additional file 9).

**Fig. 4:**
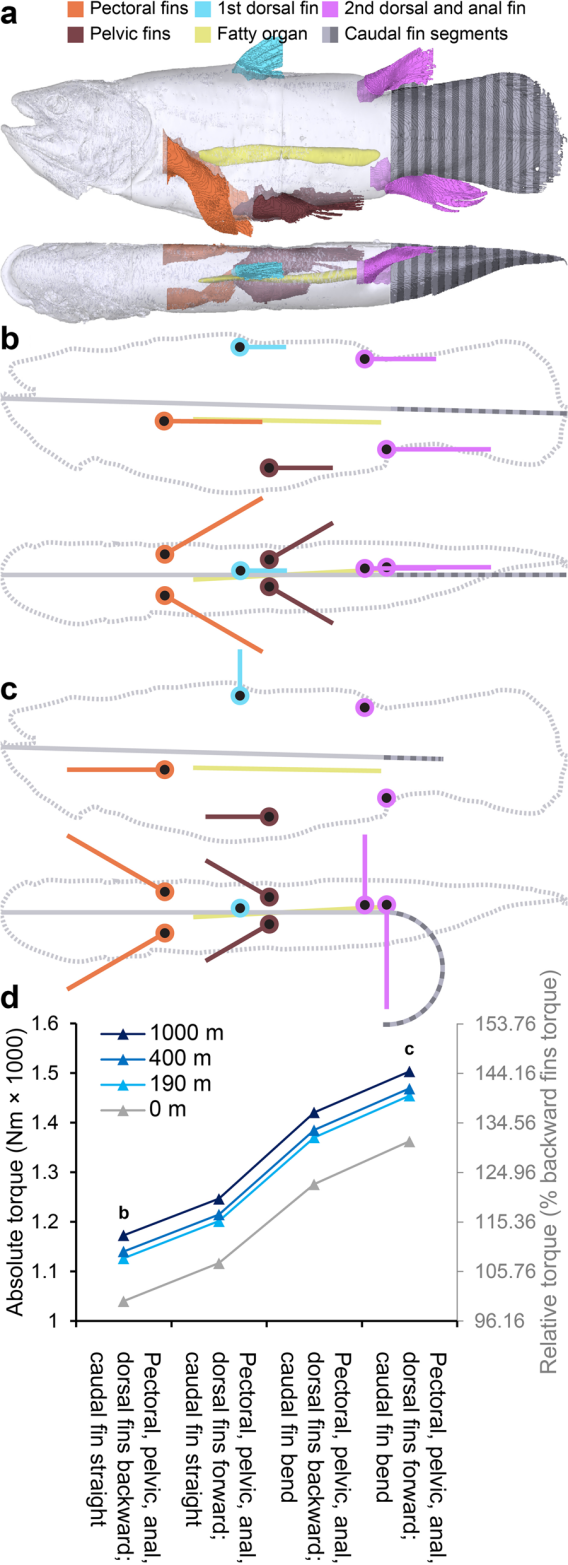
Hydrostatic balance in the coelacanth in different body postures. **a** Segmental model of the coelacanth from lateral (top) and dorsal (bottom) viewpoints consisting of 35 segments: main body, pectoral fins, pelvic fins, 1st and 2nd dorsal fins, anal fin, 26 transversal caudal fin segments, and fatty organ. **b,c** Stick figures of the segmental coelacanth model from lateral (top) and dorsal (bottom) viewpoints. Colored lines show long axis of segments with corresponding colors as in **a**. Colored circles with black centers show anchor points of fins. The coelacanth is displayed in two body postures: with straight caudal fin and remaining fins in extreme backward position (**b**) and with bent caudal fin and remaining fins in extreme forward position (**c**) (extreme fin positions according to live observation and anatomical measurements [44]). **d** Absolute (left vertical axis) and relative torque (right vertical axis). Relative to torque at the surface with straight tail and remaining fins backward at different body postures. At all modeled depths, the coelacanth can increase torque by ~ 131% by changing body posture

All the abovementioned estimates of center of gravity and buoyance are based on the coelacanth in a body posture as the studied specimen was fixated in. However, coelacanth fins are highly mobile in real life [23, 26, 27, 44], and it has been described to perform a tail bend procedure in which both the caudal and epicaudal fins are markedly bent to one side when attaining headstand postures [44]. By separating the coelacanth into 35 segments (main body, fatty organ, left/right pectoral fins, left/right pelvic fins, 1st and 2nd dorsal fins, anal fin, and 26 sequential caudal segments, see Fig. 4a and Additional file 11) and calculating center of gravity and buoyancy as well as relative mass for each segment (Table 1 and Additional file 9), the overall center of gravity and center of buoyancy in different postures can be modeled by the segmental method [45]. Since bone mineral of the paired fins and the 2nd dorsal and anal fins is distributed in the distal portion of these fins (Fig. 1a; Additional file 7), the center of gravity is positioned more distally in the appendages than the center of buoyancy. This proximodistal heterogeneity means that appendages have the ability to act as levers and decrease/increase overall torque around the pitch axis of the coelacanth when moved to different positions. The similar pattern is seen in the 1st dorsal fin although it is generally more mineralized than the remaining fins. Thus, by pointing paired, 1st and 2nd dorsal, and anal fins to a maximum forward position, the coelacanth generates a relatively larger displacements of the overall center of gravity relative to the center of buoyancy compared to pointing these fins backward, thereby increasing torque (Fig. 4b–d; Additional file 9). The mineralized hollow caudal fin rays (Fig. 1a), originally earning the coelacanth its name, result in a caudal displacement of caudal fin center of gravity to center of buoyancy (Additional file 9). By performing a semicircular tail bend, the coelacanth thus increases the center of gravity to center of buoyancy distance, thereby creating an increase in torque (Fig. 4b–d; Additional file 9). Combining the caudal tail bend maneuver with forward fin positioning, the horizontally oriented coelacanth increases torque around the pitch angle to ~ 131% relative to a backward fin, straight tail posture (Fig. 4d; Additional file 9).

### The role of the fatty organ and vestigial lung for buoyancy and hydrostatic balance

**Fig. 5:**
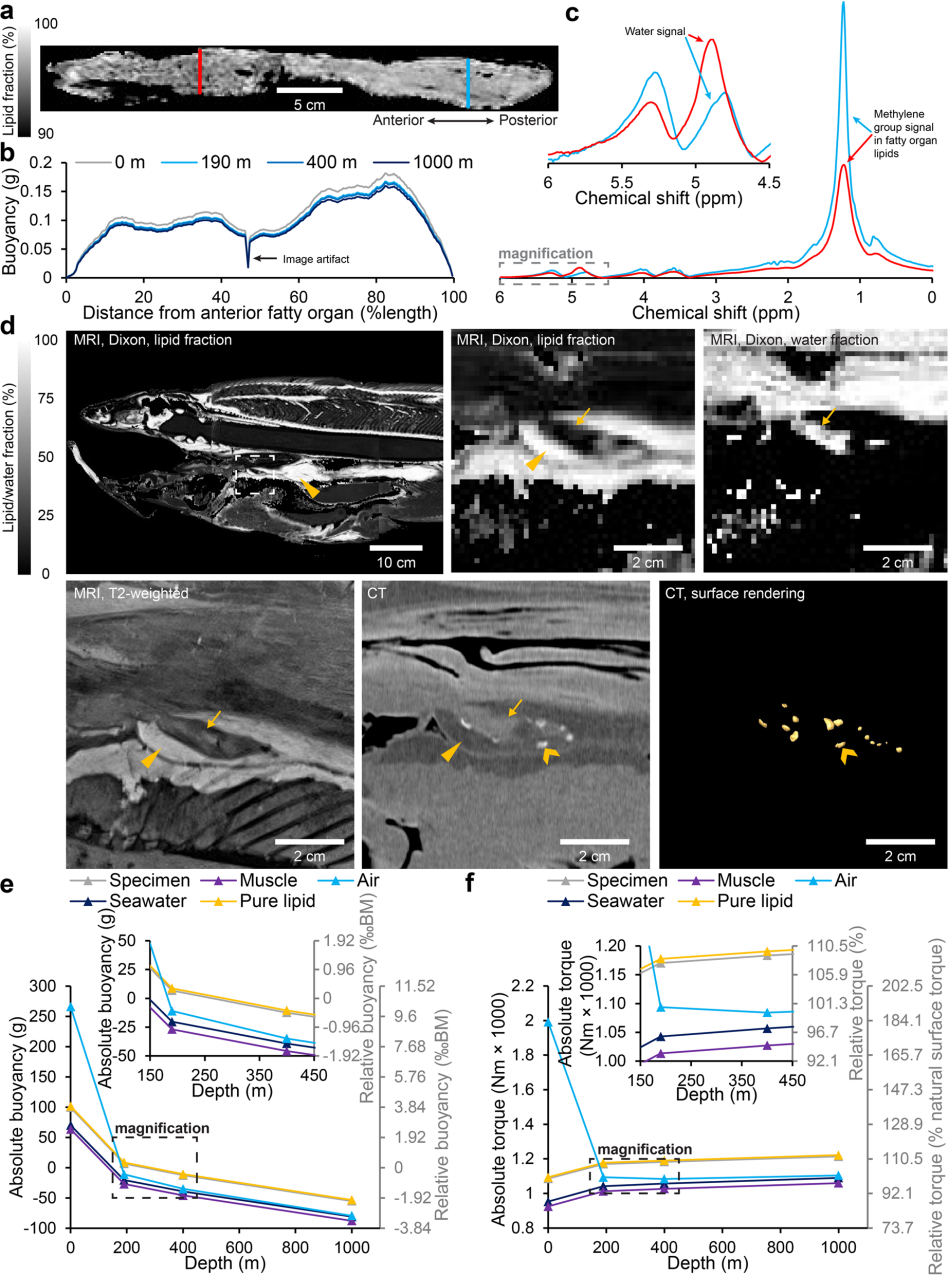
Fatty organ and vestigial lung composition and buoyancy contribution. **a** Sagittal section in the fatty organ imaged for lipid fraction with magnetic resonance imaging (MRI) using the Dixon method. Lipid fraction is visibly elevated in the caudal portion of the fatty organ. **b** Distribution of net buoyancy provided by the fatty organ (mass of tissue subtracted from mass of displaced seawater) along its long axis at four different depths in Comoran seawater. The caudal portion of the fatty organ provides more buoyancy than the cranial portion. **c** Magnetic resonance spectra acquired at the cranial (red graph and red line in **a**) and caudal (blue graph and blue line in **a**) portion of the fatty organ with the same scanning parameters (i.e., signal intensity reflects proton density). The spectrum at the caudal portion has a more pronounced methylene peak, whereas the cranial portion has a slightly more pronounced water peak, both supporting differences in lipid fraction along the craniocaudal axis of the fatty organ. **d** Tissue composition of coelacanth vestigial lung completely imbedded in the fatty organ. Low-resolution lipid (top left and top middle) and water fraction (top right) images of the cranial portion of the fatty organ (yellow isosceles triangles) containing the vestigial lung (yellow arrows). The vestigial lung contains very little lipid (see Table 1) and is instead water rich. A high-resolution T2-weighted MR image (bottom left) allows for better visualization of the vestigial lung within the fatty organ. X-ray computed tomography (CT) can be used to visualize the bony plates (yellow arrow heads) surrounding the vestigial lung (bottom middle) allowing for three-dimensional surface rendering of bony plates (bottom right). **e,f** Modeled absolute and relative buoyancy (**e**) and torque (**f**) (relative to surface torque with normal fatty organ) experienced by the coelacanth at surface, 190, 400, and 1000 m of depth in Comoran seawater if the fatty organ consisted of either seawater, muscle (or other water-rich lean tissues), pure lipid (oleyl oleate with no water content), or atmospheric air from inhalation. At the surface, an air-filled structure of similar size as the fatty organ would provide a marked increase in buoyancy and torque. However, as this air volume would compress at increasing depths under the assumption of a non-rigid wall, the lift provided by an air-filled bladder at depths would be less than that of a lipid-filled fatty organ. Magnifications in **e** and **f** contain the normal depth range of Comoran coelacanths

The fatty organ is the single largest buoyancy providing structure in the coelacanth body and in the studied specimen constitutes 0.74% of the total volume of the specimen at surface pressure and room temperature. Mapping lipid fraction using Dixon MRI revealed an uneven distribution of lipids in the fatty organ which can be observed by visualizing a medium sagittal section of the organ (Fig. 5a, notice higher lipid fraction in posterior portion of the fatty organ), and by plotting net buoyancy over the length of the organ (Fig. 5b). This observation was supported by magnetic resonance spectroscopy that revealed a more pronounced methylene group signal in the posterior portion of the fatty organ, and reversely a more pronounced water signal in the anterior portion (Fig. 5c).

The anterior portion of the fatty organ contains a vestigial lung (esophageal diverticulum) [17], which is incompletely encapsulated in mineralized lung plates (Fig. 5d). The unified structure of the fatty organ and vestigial lung has been described as a “fatty lung” [11]. In regard to the vestigial lung, this notion is unprecise since the vestigial lung in fact contains relatively little lipids (5.83%, see Table 1), which can clearly be observed in lipid fraction maps (Fig. 5d, top row).

Knowing the size and extent of the fatty organ, it is possible to model its provided buoyancy and effect on overall torque around the pitch axis if consisting of denser materials such as seawater or lean muscular tissue, or less dense materials like 100% pure lipid (oleyl oleate), or atmospheric air. At surface pressure and temperature, an air-filled structure of the size of the fatty organ would provide a substantial buoyancy lift compared to other materials (Fig. 5e) and an increased torque (Fig. 5f). However, in the depth range of Comoran coelacanths at 190–400 m as well as deeper, the ambient pressure would deflate a gas bladder without active gas secretion to a degree where it provides less buoyancy than a lipid-filled fatty organ (Fig. 5e).

### Buoyancy and hydrostatic balance in relation to feeding

**Fig. 6:**
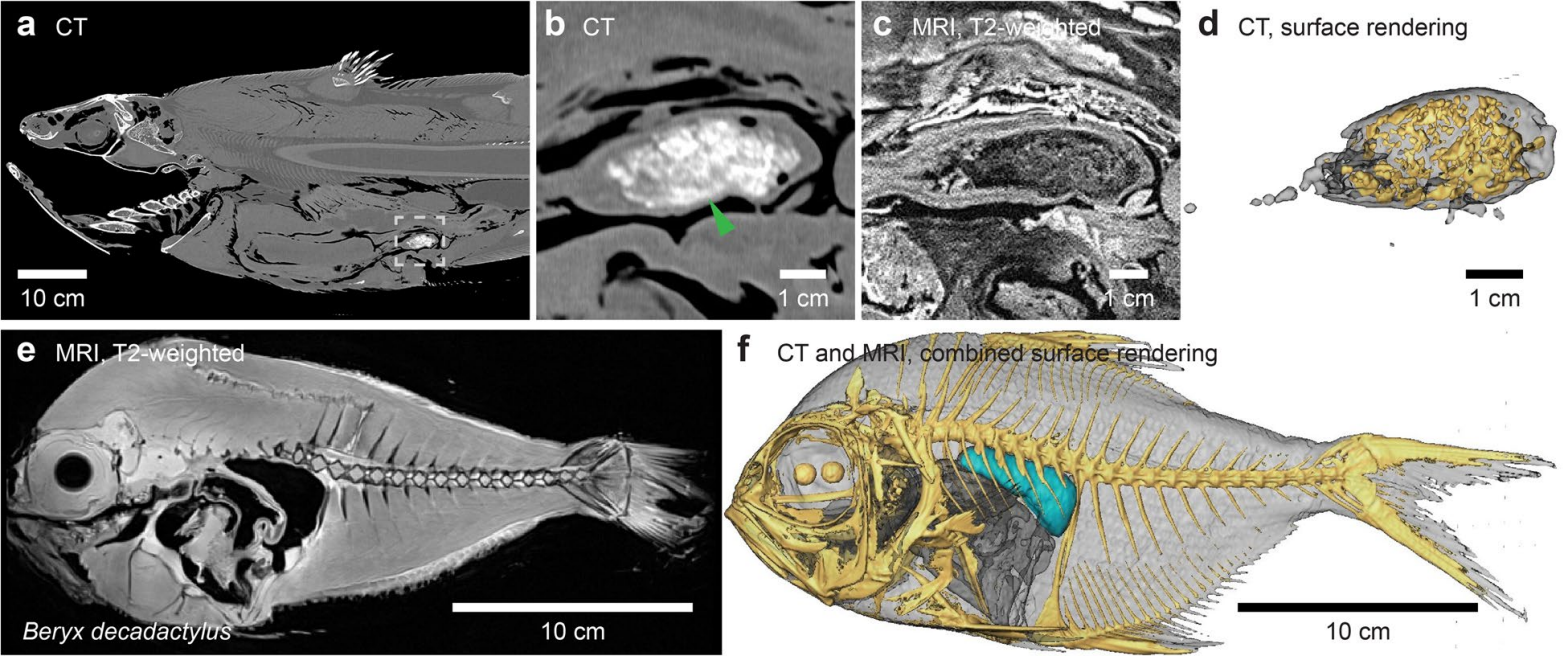
Prey items in gastrointestinal tract and prey with a swim bladder. **a–d** Mineralized remains of prey items in the distal portion of the digestive tract of the coelacanth imaged by X-ray computed tomography (CT) and magnetic resonance imaging (MRI). Dashed gray square in **a** is magnified in **b** and shown in a similar view plane using T2-weighted MRI (**c**). A three-dimensional surface rendering of the fecal matter is shown in **d**. **e,f** Sagittal T2-weighted MRI slice (**e**) and three component (skin, bones, swim bladder) model (**f**) made from CT and MRI of *Beryx decadactylus*, a known prey item of the coelacanth. This species contains an air-filled swim bladder (light blue segment in **f**) that under the assumption of neutral buoyancy displaces a volume of seawater with the same mass as the net weight of the fish (excluding the swim bladder) in seawater

A close non-invasive examination of the studied coelacanth specimen using CT and MR images revealed mineralized fecal remains in the posterior portion of the gastrointestinal tract (Fig. 6a–d; Additional files 7 and 8). The bone mineral of the feces amounts to 13.72 g, a substantial mass when the animal is close to neutral buoyancy in its natural depth range (Fig. 2d). This points to an important concern for a stationary and neutrally buoyant organism such as the coelacanth with no means of short-term regulation of buoyancy, e.g., by using an air-filled swim bladder with active gas secretion like many teleosts. Several prey species found in the stomach of dissected coelacanths possess air-filled swim bladders, e.g., *Beryx decadactylus*, *Polymixia nobilis*, and *Amioides polyacanthus* [46–49]. When ingesting a prey item with a swim bladder which is presumably neutrally buoyant at the depth of predation, the gas trapped inside the swim bladder will eventually escape upon either prey handling or digestion. Since oxygen dominates swim bladder gases in deep sea fishes [50], and the coelacanth is generally poorly equipped for oxygen uptake over the gills [51, 52], released swim bladder oxygen is likely to be dissolved relatively quickly in body fluids of the coelacanth. This leads to a net increase in coelacanth weight of exactly the mass of the seawater originally displaced by the swim bladder of the prey item. As an example, we measured swim bladder volume in a slightly smaller *Beryx decadactylus* specimen (standard length 26 cm, Fig. 6e, f) compared to what has previously been found in a smaller coelacanth than the one studied here, namely CCC 149 (body mass = 16.3 kg, total length = 92 cm) [43] containing a *Beryx decadactylus* with a standard length of 34.5 cm [49]. Swim bladder volume in the studied *Beryx decadactylus* specimen amounted to 10.28 ml equaling 10.30–10.02 g weight increase upon swim bladder collapse at the 190–400 m most frequent depth range of the Comoran population of coelacanths.

## Discussion

Anatomy and physiology are not exact sciences. Natural organisms can display large variability in terms of form and function even within species. Thus, anatomically based predictions of physiology are optimally based on observations of multiple specimens. In the case of rare animals like the coelacanth where only a little more than 300 known specimens have been procured [53], and much fewer have undergone complete analysis of body composition, it may however be necessary to lower the bar for the lowest number of specimens to be examined, if one desires to make any physiological predictions at all. Likewise, in studies of coelacanth ontogeny, it is often the case that only one specimen represents a developmental stage, e.g., [17, 18]. All measurements and predictions in this paper should be viewed in the light of them being based on a single specimen. Applying similar non-invasive quantitative imaging procedures on more specimens and on specimens from different populations of coelacanths inhabiting different depth zones would increase the level of evidence.

Predictions of buoyancy and hydrostatic balance rely on precise measurements of the distribution of lipid, lean tissue, and mineralized tissue. Overall, our non-invasive observations on the distribution of lipid-rich tissues in the coelacanth are in agreement with previous destructive measurements [33–35]. However, because it constitutes a large quantity of the body mass and thus is of importance in the overall measurement of absolute buoyancy, it should be noted that we observed a smaller lipid fraction of ventral but not dorsal muscle than has previously been reported (ventral muscle 12.77% (present study) vs. 34.1% [34], dorsal muscle 10.15% (present) vs. 7.7% [34]. All percentages in wet weight). In addition, our chemical extraction of lipids in a ventral muscle sample also yielded a lipid fraction more similar to our non-invasive measurements than literature values (4.34% of wet weight) and magnetic resonance spectroscopy supported a low level of lipids in muscular tissue (Additional file 4). This discrepancy between present and literature data on the fattiness of muscle tissue may be ascribed to large muscle samples containing lipid-rich intermuscular fat (68.13% lipid, see Table 1) being analyzed in previous destructive examinations. Although muscle sample size was not reported by Nevenzel and colleagues [34], they described that a similar protocol was used as they previously applied on *Ruvettus pretiosus* using a 276-g portion of flesh [54]. Such a large muscle sample most likely contained intermuscular fat, and the discrepancy in lipid fraction of ventral and dorsal musculature found by Nevenzel and colleagues [34] but not in the present study, could be caused by a difference in distribution of intermuscular lipid rather than an actual difference in true muscular lipid fraction (see Additional file 8). In this way, three-dimensional imaging provides an increased spatial resolution of the distribution of different tissue components. To refine the buoyancy modeling method presented in this study even further, it would be of interest to separate tissue components into more than the three dominant density classes used here: lipid, lean tissue, and bone mineral. In elasmobranchs, the balancing osmolytes urea and trimethylamine oxide also provide buoyancy [55]. Since these components are also found in high concentration in coelacanth fluids especially in the large notochordal canal [56, 57], the ability to map these chemicals using, e.g., more sensitive magnetic resonance spectroscopy or other non-invasive techniques would provide an additional refinement of buoyancy modeling. We did not observe solidification of the dominant wax ester oleyl oleate at the relevant temperatures used in this study; however, it would also be of interest to investigate this for the mix of wax esters found in coelacanth lipid deposits [34], since phase shift can cause rapid density shifts and thus alter overall buoyancy as suggested in cetaceans [38, 39]. 

Depth regulation is of major importance in aquatic organisms, and several mechanisms to maintain a desired depth, ranging from active hydrofoiling to provide lift to passive mechanisms to control absolute density of the body, have been extensively studied in several vertebrates, e.g., [36, 55, 58–64]. The relative buoyancy of an organism provides useful and practical (can be measured in field and nautical conditions) information about the potential employment of passive mechanism to adjust buoyancy. However, this measure obtained at the surface, potentially at different water and animal temperatures, water salinity, and definitely at a reduced pressure, must always be considered a proxy of actual buoyancy in the organism’s own habitat, since this measure can only be calculated by knowing the chemical components of the organism and how their density change at depth [39, 59]. Since early observations on living coelacanths, the general notion has been that the coelacanth is neutrally buoyant or close to and remains hovering and stable in the water with little effort (see Additional file 1 for historical observations). This is sensible given that the coelacanth has been estimated to have a metabolic rate and oxygen extraction capacity in the lower range of all known vertebrates [42, 51, 52], and it is therefore not unlikely that it has experienced a strong selection pressure to reduce energy expenditure. Our data on buoyancy support this notion and there is a clear match between the zone of neutral buoyancy and the habitat depth of the Comoran population of coelacanths (Fig. 2d). Additionally, we found the coelacanth to be in close-to-perfect hydrostatic balance although the center of gravity is positioned slightly anterior and very slightly dorsal to the center of buoyancy (Fig. 3) resulting in a small torque around the pitch axis when the animal is in a horizontal orientation. This torque can be increased if the animal changes posture, moving paired and unpaired fins to a maximum forward facing position and bending the caudal fin (Fig. 4). The coelacanth has been observed to perform headstand maneuvers lasting for minutes as it drifts across the substrate with the head facing downwards placing the narrowly focused electrosensitive rostral organ in close proximity to the seabed which may maximize changes of benthic prey capture [27, 31, 44, 65]. In the words of Fricke and Hissmann [44] “the slow lateral curving of the caudal and epicaudal fins is the most obvious pattern of the sequence” leading to a headstand. This curving of the posterior body would increase torque around the pitch axis (Fig. 4), thereby aiding the shift from a normal orientation to a headstand. Even when the coelacanth restraightens the caudal fin during the headstand, the anterior position of the center of gravity to center of buoyancy will ensure that it can drift in this abnormal posture with minimal need for movements of balance adjustments, which potentially increases the changes to sneak up on prey.

For any neutrally buoyant organism, feeding on other prey items that are not neutrally buoyant, or that lose this ability as an air bladder is punctured, may represent a concern. Here we showed that a larger bodied coelacanth ingesting a smaller bodied prey item with a swim bladder than described in the literature [49] would in fact loose ~ 10 g of lift (Fig. 6). Until the heavy contents of a prey item have been digested, this would potentially result in an extra energy expenditure of the coelacanth to keep station.

The presence of a calcified bladder in fossil coelacanths was discovered early on [66], but it was only within the previous decade that this structure was identified as a lung [67]. Later, small bony plates surrounding the esophageal diverticulum of extant coelacanths (see Fig. 5d) were used to recognize that this structure constituted the homologous calcified lung [17, 68], thereby questioning the fatty organ which has previously been referred to as “fatty lung” [11], “swimbladder” [69], or “modified lung” [70] to be a part of the pulmonary complex altogether [68, 71]. On the other hand, it has also been suggested that instead of representing the only sarcopterygian with an unpaired lung (*Neoceratodus forsteri* also has a single lung, the right, but an anlage for the left is formed early during ontogeny [72]), it may be so that the dual presence of a fatty organ and a vestigial lung in the coelacanth may in fact indicate a paired lung homolog in which one branch became lipid filled to provide buoyancy in extant coelacanths and one regressed to the present vestigial lung [73, 74]. Without any presumption about the origin of the fatty organ nor the assumption that this organ in the extant coelacanth represents a similar volume as the calcified lung in extinct forms, we modeled the effect on buoyancy and hydrostatic balance in the extant coelacanth if this organ was constituted of other substances than lipid (Fig. 5e, f). Most notable was the effect on buoyancy if the fatty organ consisted of atmospheric air (Fig. 5e). At shallow depths, an air-filled organ of the size of the fatty organ would provide substantial lift, potentially alleviating the need for additional lipid resources to provide buoyancy. However, at the habitat depth of the extant population of Comoran coelacanths, an air bladder of the modeled size would deflate to such a degree that a lipid-filled fatty organ would in fact provide more lift (Fig. 5e) potentially pointing to the selective force that drove lipid accumulation in the coelacanth incapable of oxygen secretion (high hemoglobin affinity for oxygen and limited Root effect [75]) as well as in other moderate and deep sea fishes [58, 60]. It should however be noted that the volume of the fatty organ only takes up 0.74% of total body volume in the extant coelacanth whereas most teleost fishes in the neritic zone possess a swim bladder of up to 5% of total body volume [76] (most likely owing to the fact that average fish tissue including both lean tissue, lipids, and bones is 5% denser than seawater [70]). An air-filled bladder of this size would provide increased buoyancy if present in the coelacanth, even if compressed at depth. If possible, it would be of interest to compare calcified lung size to body size in fossil coelacanths to infer information about the potential role of this organ as a buoyancy control structure and if it was lipid- or air-filled. In agreement with other reports [77, 78], we found the vestigial lung of *Latimeria* to possess limited amounts of lipid (Table 1) pointing to a non-buoyancy-specific function of this structure unless it was air-filled in extinct species. The notion that bony walls of the lung in some extinct coelacanths would provide both rigidity and protection against hydrostatic pressure and better containment of gas [70, 71], thereby aiding its function as a buoyancy device, must be viewed with some reservation, at least concerning the possibility for extinct coelacanths to perform vertical migrations aided by this putatively rigid buoyancy structure. To withstand hydrostatic pressure at greater depths than shallow water conditions, a calcified lung must be considerably mineralized, and since mineralized bony material is a major source of negative buoyancy, this may directly counteract any potential benefits of an incompressible air-filled buoyancy structure. Another possibility is that the wall of the calcified lung in extinct coelacanths was not completely rigid, but rather consisted of large connected plates moving over each other to accommodate certain volumetric changes [67]. Again, in the view of the calcified lung as a potential buoyancy providing structure, this must be viewed as a trade-off between keeping a gas volume in a semi-rigid structure to provide lift and keeping the weight of the bony walls down.

Female coelacanths are larger than males [43], and females are generally observed at larger depths [4, 42]. It would be of interest to repeat the non-invasive examinations presented here in female specimens to study how this may influence composition of mineralized and fatty tissue. Related to this, a potentially useful application of non-invasive bone mineral and lipid mapping is in the prediction of habitat depth of newborn coelacanths. It remains unresolved where gravid females give birth and at which depth neonatal coelacanths reside, although based on a record dive unusually performed at daytime of a pregnant female tracked with acoustic telemetry to 698 m [4], it has been speculated that coelacanths may be born deeper than their usual habitat [31]. Late-term (CCC 162.1–26) and a juvenile coelacanth (CCC 94) are represented in museum collections [43], and some have undergone non-invasive imaging for morphometric and ontogenetic purposes [18, 79]. It would be of interest to employ quantitative procedures as described here and use bone mineral calibrated micro-CT and lipid calibrated micro-MRI data to model buoyancy at different depths for these smaller specimens. If adapted to a deeper habitat, the increased compression of tissue lipids relative to that of lean tissue and bone mineral would predict neonatal coelacanths to contain relative less bone mineral or relatively more lipids or both compared to adult coelacanths to remain neutrally buoyant in the deep.

Despite continuous contributions from direct and indirect observations from, e.g., submersibles, baited traps, remotely operated underwater vehicles, and black water scuba diving [80, 81], the behavior and physiology of many deep sea fishes remain unknown. The non-invasive method presented here supplements collection-based research on rare and otherwise unavailable species. As demonstrated here, information about buoyancy can be crucial for our understanding of habitat shift, migrations, spawning sites, etc. of rare iconic species. Combined with oceanographic data, they may help explain the evolutionary history (incl. extinctions) and climate change-related shifts in distributions and fish faunas. Similar detailed information would be valuable for a large number of commercial and threatened species (e.g., *Anguilla anguilla* and *Rhincodon typus*).

## Conclusions

In conclusion, we have used non-invasive imaging on a single coelacanth specimen to find support for that the extant coelacanth is neutrally buoyant and close to hydrostatic stability in its natural habitat. This is attributable to a lightly mineralized skeleton and large lipid deposits, especially in the fatty organ that functions better as an organ of buoyancy than a similar sized air bladder at the coelacanth’s habitat depth. Food intake of prey items with swim bladders can compromise neutral buoyancy. Applying similar non-invasive methods on a larger number of specimens of both sexes and different age groups can provide additional important information regarding depth adaptations in the extant species of coelacanths. Finally, alluding to the acrobatics that neutral buoyancy and hydrostatic stability confer on the coelacanth, it may be appropriate to cite Professor Hans Fricke, one of few who have observed the extant coelacanth in its natural environment, on an observation made during a dive in his submersible *Jago*: “Ein Tier stand vor der Höhle auf dem Kopf—prachtvoll, schön, stark und gesund. Alle Tiere schienen in ausgezeichnetem Zustand zu sein. Ein anderes stand senkrecht auf seinem Schwanz, den Kopf nach oben. Auch in dieser Körperlage hielt es sich mit Leichtigkeit in stabiler Position” [31].

## Methods

### Specimen information

The imaged coelacanth specimen is an adult male West Indian Ocean coelacanth, *Latimeria chalumnae* Smith, 1939, CCC 23, fished at 250 m of depth and 700 m off the coast between Iconi and Moroni, Grande Comore (Ngazidja) at 1:00 h on June 23, 1960. Total length was 130 cm. The specimen was reportedly only preserved in 70% ethanol without the use of formaldehyde [43]. Since this was unconventional for the era, this was tested using a formaldehyde assay test kit (see chemical analysis section below). The left side of the abdomen has been opened with a longitudinal cut, and in the head, one gill arch has been bent; however, all inner organs remain intact (see Additional files 7 and 8). The specimen was received as a gift on June 16, 1962, at the Zoological Museum, University of Copenhagen, Denmark, from the Muséum National d'Histoire Naturelle, Paris, France. It has been on display at Danmarks Akvarium and later on the Natural History Museum of Denmark, but is currently stored in the collection (catalog number ZMUC P1112). For all imaging procedures, the coelacanth specimen was placed on its left side (same position as it was presumably fixed in); however, to better display chemical content values in a typical snout-to-tail/left-to-right reading direction, datasets were flipped along the horizontal axis to achieve this. This has no effect on any calculations. Additional species were from the collection of the Natural History Museum of Denmark: *Neoceratodus forsteri* (Krefft, 1870) (ZMUC Journal# 4), *Lepidosiren paradoxa* Fitzinger, 1837 (ZMUC P1124), *Protopterus annectens* (Owen, 1839) (ZMUC P1122), *Dissostichus eleginoides* Smitt, 1898 (ZMUC P63215), *Hoplostethus atlanticus* Collett, 1889 (ZMUC P40335), *Spectrunculus grandis* (Günther, 1877) (ZMUC P77701), *Myctophum humboldti* (Risso, 1810) (ZMUC P2397221), *Thalassobathia pelagica* Cohen, 1963 (ZMUC P77853), *Hexanchus griseus* (Bonnaterre, 1788) (uncat.), *Typhlonus nasus* Günther, 1878 (ZMUCP77447), *Solivomer arenidens* Miller, 1947 (ZMUC P2341238), *Neopagetopsis ionah* Nybelin, 1947 (ZMUC P7641), *Aeoliscus strigatus* (Günther, 1861) (ZMUC P39373), *Beryx decadactylus* Cuvier, 1829 (ZMUC P4013), and a beached *Mola mola* (Linnaeus, 1758) (uncat.). Nine specimens of *Sparus aurata* Linnaeus, 1758 (uncat.) were purchased fresh and ungutted from a local fish market for the chemical analysis and validation of methods for bone mineral and lipid content measurements. Putative time calibrated phylogeny of extant non-tetrapod sarcopterygians was acquired from TimeTree (http://timetree.org/ accessed on June 22, 2021).

### Imaging

For lipid/water mapping, MRI was performed on an Siemens Magnetom Skyra system equipped with a row of coils in the scanner table and two Siemens Body 18 and one Flex Large 4 surface coils using a Dixon flash 3D sequence with the following parameters: field strength: 3 T, repetition time = 5 ms, echo time = 1.23 ms, flip angle = 10°, field-of-view = 423.3 × 500.0 × 318.1 mm^3^, spatial resolution 1.42 mm isotropic, number of averages = 3, acquisition time = 1.4 h pr. scan. Four scans were performed to image the entire specimen, and these were subsequently concatenated using ImageJ 1.50e. An ethanol dilution series of 0–100% v/v ethanol in distilled water in steps of 10% v/v was imaged using the same Dixon flash 3D sequence for subsequent lipid signal correction of the effect of tissue ethanol. The same MRI system quipped with a Flex Small 4 surface coil was used to acquire high-resolution images of the vestigial lung and stomach object using a T2-weighted spin echo sequence with the following parameters: field strength: 3 T, repetition time = 1000 ms, echo time = 139 ms, field-of-view = 134 × 134 × 67.2 mm^3^, spatial resolution 0.35 mm isotropic, number of averages = 4, acquisition time = 4 h pr. scan.

Quantitative CT was performed on the coelacanth specimen using a Siemens Somatom Definition Dual Energy system with the following parameters: X-ray tube voltage = 120 kVp, X-ray tube current = 428 mA, integration time = 1000 ms, field-of-view = 102.0 × 102.0 × 207.6 mm^3^, spatial resolution = 0.2 mm isotropic, convolution kernel = B45s, acquisition time = 90 s pr. scan. A total of 53 tile scans of small portions of the coelacanth specimen were performed and these tiles were subsequently combined using ImageJ 1.50e creating a dataset covering the entire specimen at a high resolution. A Mindways QCT Pro bone mineral calibration phantom placed underneath the specimen was used to calibrate X-ray attenuation values to bone mineral density (mg/mm^3^ equivalent aqueous K_2_HPO_4_). An ethanol dilution series of 0–100% v/v ethanol in distilled water in steps of 5% v/v was imaged using the same imaging protocol together with a sample of the preservation liquid for the coelacanth specimen. Additional CT scans were performed on species with relevance for the study on either the same CT system with similar parameters but varying resolution depending on sample size (*Protopterus annectens*, 0.39 × 0.39 × 0.6 mm^3^; *Dissostichus eleginoides*, 0.94 × 0.94 × 0.6 mm^3^; *Neopagetopsis ionah*, 0.5 mm isotropic; *Mola mola*, 0.97 × 0.97 × 0.6 mm^3^; *Sparus aurata*, 0.6 mm isotropic) or another CT system (Toshiba Aquillon Prime SP) with similar parameters but varying resolution depending on sample size (*Neoceratodus forsteri*, 0.6 × 0.6 × 0.5 mm^3^; *Lepidosiren paradoxa*, 0.2 mm isotropic; *Hoplostethus atlanticus*, 0.4 mm isotropic; *Spectrunculus grandis*, 0.3 mm isotropic; *Thalassobathia pelagica*, 0.3 mm isotropic; *Hexanchus griseus*, 0.6 × 0.6 × 0.5 mm^3^; *Typhlonus nasus*, 0.3 mm isotropic; *Beryx decadactylus*, 0.5 mm isotropic) or for the smaller species (*Myctophum humboldti*, *Solivomer arenidens*, *Aeoliscus strigatus*) a high-resolution clinical extremity CT system (Scanco Medical XtremeCT; Scanco, Brüttisellen, Switzerland) with the following parameters: X-ray tube voltage = 59.4 kVp, X-ray tube current = 119 µA, integration time = 132 ms, field-of-view = 70 × 70 × 150 mm^3^, spatial resolution = 0.082 mm isotropic, acquisition time = 1.5 h pr. scan.

Photogrammetry of the right side of the coelacanth specimen was performed using 48 photos at 6000 × 4000 px^2^ resolution captured at different angles using a Canon DSLR camera. Photos were assembled to a three-dimensional model using Autodesk Recap Photo.

### Nuclear magnetic resonance spectroscopy

^1^H nuclear magnetic resonance spectroscopy was performed using both single-voxel spectroscopy at selected organs (fatty organ, post ocular, muscle, notochord) as well as chemical shift imaging across the entire coelacanth body at slices spaced 51 mm apart (16 anteriormost slices) or 102 mm apart (2 posteriormost slices). The same imaging system as for MRI, a Siemens Magnetom Skyra system equipped with body and surface coils, was used for both single-voxel spectroscopy and chemical shift imaging. Single-voxel spectroscopy was performed using a PRESS sequence with the following parameters: field strength: 3 T, repetition time = 2000 ms, echo time = 33 ms, bandwidth = 1200 Hz, flip angle = 90°, voxel size = 10 × 10 × 10 mm^3^, number of averages = 128, acquisition time = 256 s. Acquisition was performed both with and without water suppression. Chemical shift imaging was performed using a chemical shift imaging spin echo sequence with the following parameters: field strength: 3 T, repetition time = 1500 ms, echo time = 35 ms, bandwidth = 1200 Hz, flip angle = 90°, field-of-view = 180 × 180 × 10 mm^3^, spatial resolution 11.25 × 11.25 × 10 mm^3^, number of averages = 4, acquisition time = 1536 s. Acquisition was performed both with and without water suppression. At each spectroscopy step, an anatomical reference image was acquired using the Dixon sequence described above.

For prediction of ^1^H nuclear magnetic resonance spectra for ethanol and oleyl oleate, molfiles of each molecule were downloaded from PubChem (https://pubchem.ncbi.nlm.nih.gov), imported into nmrdb (https://www.nmrdb.org) and their spectrum were simulated using parameters as similar as for the magnetic resonance spectroscopy acquisition: spectrometer frequency = 100 MHz, line width = 1 and 5 Hz, range = 0–6 ppm. Labile protons, e.g., OH, are not predicted in nmrdb, thus no prediction of the hydroxy group in ethanol was expected.

### Chemical and physical analyses of tissue

It was imperative to keep the coelacanth specimen intact; thus, in addition to the non-invasive imaging procedures undertaken on the specimen in this study, only a small ventral muscle sample was taken for chemical analysis.

Lipid extraction was performed to measure lipid fraction of the ventral muscle sample of the coelacanth specimen using a chloroform-free lipid extraction kit validated to perform equally well as the Folch method of lipid extraction (ab211044, Abcam). The extracted amount of lipid of the ventral muscle sample of the coelacanth was insufficient to perform temperature-dependent density and solubility testing; thus, pure oleyl oleate (Sigma-Aldrich O3380), the dominant wax ester found in coelacanth tissue [34], and *Hoplostethus atlanticus*, extracted swim bladder lipid which is dominated by wax esters quite similar to those of the coelacanth [41], were taken as proxies for physical behavior of lipids found in the coelacanth. Average density of oleyl oleate and the extracted *Hoplostethus atlanticus* swim bladder lipid at different temperatures was measured by adjusting lipid temperature on a thermoshaker and then pipetting out and weighing subsamples of known volume. Measurements were pressure adjusted by assuming similar physical behavior as wax esters found in the marine copepod *Calanus plumchrus* Marukawa, 1921 that has been measured in a range of deep sea relevant pressures [82]. Temperature, salinity, and depth information in the habitat of the coelacanth was taken from reference values [42]. Comoran seawater density at varying temperature, salinity and pressure was calculated according to standard procedures [83].

Tissue ethanol and formaldehyde concentration was measured in the coelacanth muscle sample with colorimetry kits (Formaldehyde: K-FRHYD 01/21 and Ethanol: K-ETOH 08/18, both produced by Megazyme) using catalyst-mediated formation of the reduced form of nicotinamide adenine dinucleotide that can be measured by the increase in absorbance at 340 nm. To compare tissue formaldehyde concentration in the coelacanth with concentrations found in fish tissue after different preservation methods, the nine *Sparus aurata* specimens were preserved using three different methods: physical preservation by freezing (*n* = 3), chemical preservation using phosphate buffered formalin (4% v/v formaldehyde) fixation followed by storage in 70% v/v ethanol (*n* = 3), or another method of chemical preservation by direct immersion in 70% v/v ethanol followed by storage in 70% v/v ethanol together with formalin fixed specimen (*n* = 3). Tissue formaldehyde concentration was measured in muscle samples from these physically and chemically preserved *Sparus aurata* specimens using the same colorimetry kits as for the coelacanth sample.

The effect of preservation method on bone mineral content was measured by non-destructive quantification using quantitative CT on the nine *Sparus aurata* specimens before and after preservation and thereafter validated to actual amount of bone mineral using ashing (12 h at 90 °C then 72 h at 580 °C) of homogenized samples. The effect of preservation method on lipid content was measured by performing lipid extraction on the nine *Sparus aurata* specimens after homogenization.

Solubility of pure oleyl oleate, extracted swim bladder lipid of *Hoplostethus atlanticus* and extracted full body lipid of *Sparus aurata* (in contrast to the wax esters of coelacanth and *Hoplostethus atlanticus*, lipids of *Sparus aurata* are dominated by triglycerides and phospholipids [84], thus representing other types of lipids also found in the coelacanth although at a lower concentration than wax esters [34]), in 70% v/v EtOH at varying temperatures from 20 to 40 °C in steps of 5 °C was measured by incubating lipids with 70% v/v EtOH in separate containers on a thermoshaker using 3 h of shaking (500 RPM) and 1 h of rest pr. temperature level. Samples of the lipid mixtures were drawn at each temperature and dried (6 h at 78 °C and 12 h at 99.9 °C). Dissolved lipid content of coelacanth, *Hoplostethus atlanticus* and *Sparus aurata* 70% v/v ethanol storage solutions was measured by drying (6 h at 78 °C and 12 h at 99.9 °C) a portion of each storage solution and subsequently performing lipid extraction to remove debris and dissolved proteins.

### Modeling of buoyancy and hydrostatic balance

Acquired CT and MRI data in 16-bit color depth was initially reformatted into 32 bit for precision of subsequent calculations. Thereafter, CT data outputted in Hounsfield units was converted into bone mineral density using the calibration phantom and lipid- and water-specific images of the MRI data were converted into lipid and water fraction images. Quantitative CT data was then adjusted for the effect of ethanol preservation on measurements of bone mineral content, and MRI data was adjusted for the effect of ethanol preservation on measurements of lipid content. Both datasets were cropped in each dimension to only include specimen voxels and background pixels were removed using the ImageJ Threshold plugin. Both the CT and the MRI datasets were segmented into 35 segments: main body, fatty organ, left/right pectoral fins, left/right pelvic fins, 1st and 2nd dorsal fins, anal fin, and 26 sequential caudal segments. For each segment, average proportions of bone mineral, lipid, and water (lean tissue) was measured in every slice in the transversal, sagittal, and coronal plane, respectively, using the ImageJ multi-measure function. While both CT and MRI datasets where isotropic and covered the entire coelacanth specimen, image resolution was 357.9 times higher in the CT dataset (0.2 mm vs. 1.42 mm in each spatial dimension), thus resulting in 7.1 times the number of slices spanning the specimen in each spatial dimension. To allow for the combination of bone mineral content measurement from CT with lipid and water content from MRI, each slice measurement of bone mineral content was scaled by the number of slices in the same spatial dimension of the MRI dataset and then binned by the number of slices in the CT dataset, thus resulting in slice measurements in equal numbers.

To measure absolute mass of bone mineral, lipid, and lean tissue for each segment of the specimen, slice-wise proportions of each substance was multiplied by the modeled density of the substance at the given depth (0, 190, 400, and 1000 m). Lean muscle density [58] was adjusted to pressure and temperature by the same factor as sea water. To measure absolute buoyancy for each segment, the volume displaced by the segment was multiplied by density of Comoran sea water at the given depth.

To measure center of gravity (*X*_*COG*_) and center of buoyancy (*X*_*COB*)_ for each segment of the specimen in each anatomical axis, moments which is mass (*m*) times distance (*x*) was calculated for each slice (*i*) and combined in each axis (transversal, sagittal, coronal) using the equations:

$${X}_{COG}=\frac{\sum_{i=1}^{N}{(m}_{lipid,i}{+ {m}_{lean,i}+{ m}_{bone mineral,i}) \times x}_{i}}{M}$$

$${X}_{COB}=\frac{\sum_{i=1}^{N}{m}_{displaced seawater,i}{ \times x}_{i}}{M}$$

where *N* is the total number of slices and *M* is the total mass of the segment. The mass of displaced seawater was calculated as the density of Comoran seawater at the given depth multiplied by the volume of tissue when corrected for volume changes due to pressure and temperature. After relative mass and the three-dimensional location of the center of gravity and center of buoyancy of each segment was determined, the overall center of gravity and center of buoyancy of the entire specimen was calculated using the segmental method by dividing the sum of segment moments about each axis by the total relative body weight [45] (for detailed calculations see Additional file 9).

Modeling center of gravity and center of buoyancy shift at different body postures was performed as above but with the addition of adjusting the coordinates of the center of gravity and center of buoyancy to a given fin angle using trigonometric functions. Maximum fin angle relative to the coelacanth’s long axis were taken from [44, 85]: pectoral and pelvic fins: ± 120° mobility in the anterior–posterior direction, second dorsal and anal fin: 90° lateral mobility, first dorsal fin: 90° dorsoventral mobility. Caudal and epicaudal fin was assumed to be able to bend to a semicircular shape based on available photographic material [28, 44]. Torque (*τ*) was calculated from the force of magnitude (*F*) applied at a distance (*r*, *X*_*COG*_ to *X*_*COB*_ distance) from the axis of rotation (center of gravity) in an orientation where the arm makes the angle (*θ*) with the respect to the line of action of the force using the equation:

$$\tau =r\times F\times \mathit{sin}\uptheta$$

## Supplementary Information

**Additional file 1.** Primary and secondary historic observations onbuoyancy and hydrostatic balance in both species of *Latimeria* (only known extant genus of coelacanths) and observationson lipid content. Observations are arranged chronologically if exact orapproximate time of observation is known. The sources of observations areseparated into scientific for peer-reviewed scientific journals ornon-scientific for popular science magazines or books, and primary or secondarydepending on if the source was the observer him/herself or a secondary author.The extracted information on buoyancy and hydrostatic balance is presented anda relevant citation is provided if present (most relevant part is underlined).Non-English citations are provided both in original language and in atranslated version.

**Additional file 2.** Additional results and discussion.

**Additional file 3.** Chemical analyses on the effect of ethanolpreservation on actual bone mineral and lipid content and measurement errorusing CT and MRI. **a**, Formaldehyde(Form.) concentration in homogenized tissue samples of *Sparus aurata* (*S. aur*) indifferent states (fresh unfixed specimens, non-formaldehyde fixed but ethanol(EtOH) preserved specimens stored together with formaldehyde fixed specimen for11 months, and formaldehyde fixed and EtOH preserved specimens) compared to thetissue of the studied coelacanth (*L. cha*).The coelacanth in this study contains a similar amount of residual formaldehydein the tissue as formaldehyde fixed *S.aur* specimens. **b**, Hounsfieldunit of solutions with increasing concentrations of EtOH. **c**, Lipid fraction error from Dixon MRI of lipid free solutions withincreasing concentration of EtOH. A tissue concentration of EtOH of 37.38% v/vas measured in the coelacanth in this study results in a 4.75% error in thelipid fraction reading. **d**,Solubility in 70% v/v EtOH of oleyl oleate (most prevalent wax ester incoelacanth), swim bladder lipid of *Hoplostethusatlanticus* (*H. atl*) and full bodylipids of *S. aur *as a function oftemperature and lipid concentration in old storage medium. **e**, Relative bone mineral content (BMC) in *S. aur* specimens as estimated by quantitative x-ray computed tomography(qCT) before any fixation and after either direct EtOH preservation orformaldehyde fixation followed by EtOH preservation compared to measured BMCfrom ashing. Values are normalized to average BMC of each group in the freshunfixed state. Preservation in 70% v/v EtOH results in an underestimation(60.35%) of actual BMC due to the decreased x-ray attenuation in EtOH comparedto water (see **b**). Fixation andpreservation does not have significant effect on BMC of specimens (nosignificant difference for all groups between “No chemical fixation” and“Ashed”). Colored dashed lines are shown between categorical measurement pointsto highlight the paired nature of samples.**f**, Relative lipid content(LC) in *S. aur* specimens in differentstates (fresh unfixed specimens, non-formaldehyde fixed but EtOH preservedspecimens, and formaldehyde fixed and EtOH preserved specimens). Fixation andpreservation does not have significant effect on LC of specimens. Lettering (A,B, C) above graph points denotes significant group differences in all panels,*p*-values are provided in the text. All error bars represent standard deviation.

**Additional file 4.** Magnetic resonance spectroscopy in the body ofthe coelacanth.** a-b**, Predictednuclear magnetic resonance spectra of ethanol (EtOH) (**a**) and the dominant wax ester, oleyl oleate (**b**), in the coelacanth. Spectra were prepared using both a linewidth of 1 Hz (red) to show individual peaks and 5 Hz (black) to betterrepresent results of low field strength spectra acquired in the specimen. **c**, Combined plot of acquired spectra inpure water, ethanol, fatty organ (caudal position, see Fig. 5a), post ocular,muscle, notochord. Since spectra were acquired with the same acquisitionparameters, the absolute values can be used as a proxy for proton density, i.e.,proton density of the 4.7 ppm water signal in pure water (light blue) is largeas expected and so is the proton density of methylene groups of wax esters inthe fatty organ (yellow). **d-h**,individual spectra of ethanol (**d**),muscle (**e**), notochord (**f**), post ocular (**g**), and fatty organ (**h**).While muscle and notochord are dominated by water and ethanol signals, postocular tissue and especially the fatty organ is more dominated by the lipid signal.

**Additional file 5.** Distribution of bone mineral in some species ofosteichthyes and chondrichthyes with reported neutral or near neutral buoyancyor pelagic or deep-sea lifestyles that would suggest so.** a**, Volume rending of bone mineral density (BMD) map of thecoelacanth, ten species of osteichthyes and one species of chondrichthyes.Species are ranked according to the similarity of bone mineral distributionrelative to the coelacanth (*Dissostichuseleginoides* most and *Aeoliscusstrigatus* least similar). *Dissostichuseleginoides*, *Neopagetopsis ionah*,*Mola mola*, *Aeoliscus strigatus* and *Hexanchusgriseus* are reportedly neutrally or close to neutrally buoyant [21, 86–88]. *Hoplostethus atlanticus*, *Myctophum humboldti* and *Solivomer arenidens* contain a lipidfilled swim bladder proposed to have a similar function as the fatty organ ofthe coelacanth [58], whereas *Dissostichuseleginoides*, *Neopagetopsis ionah*,*Mola mola* and *Hexanchus griseus* don’t contain any swim bladder. *Spectrunculus grandis*, *Thalassobathia pelagica* and *Typhlonus nasus* may conduct similar headdown hunting maneuvers similar to what is observed in the coelacanth. Note thatBMD color bar for *Mola mola* spans 0 –0.5 mg/µl. **b**, Distribution of bonemineral content (BMC) fraction of total BMC along the total length (TL) in thespecies above the panel (the black graph for the coelacanth, *L. cha*, is present in both panels forcomparison). Dashed gray squares in the most caudal 33% portion of the graphsare magnified in the insertions to highlight species with similarly mineralizedcaudal fins as the coelacanth.

**Additional file 6.** Distribution of bone mineral in some species ofosteichthyes and chondrichthyes with reported neutral or near neutral buoyancyor pelagic or deep-sea lifestyles that would suggest so. The table containsmeasurements and calculations leading to Additional file 5b-c. For eachspecies, a bone mineral density calibrated quantitative x-ray computedtomography scans was used to separate the specimen into 835 separate slice forwhich the bone mineral content (BMC) and slice fraction of total BMC wasmeasured. Total BMC per specimen can be found in column AH.

**Additional file 7.** Slice video of bone mineral density from x-raycomputed tomography scan. The coelacanth specimen is sequentially sliced in thetransversal, sagittal and coronal axis. Slice thickness is 0.6 mm in eachdimension (×3 binned from original data with 0.2 mm isotopic resolution). Slicerate is 30 slices/s.

**Additional file 8.** Slice video of lipid (transversal: left,sagittal: top, coronal: top) and water fraction from magnetic resonance scan.The coelacanth specimen is sequentially sliced in the transversal, sagittal andcoronal axis. Slice thickness is 1.42 mm in each dimension. Slice rate is 30slices/s.

**Additional file 9.** Calculations of buoyancy, center of gravity andcenter of buoyancy for individual body segments (main body, fatty organ,pectoral fins, pelvic fins, dorsal fins, anal fin and 26 caudal fin segments)and combined. First tab contains combined measurements and all calculationsleading to Figs. 2d, 3e, f and 4d. Second tab contains calculations leading toTable 1. Subsequent tabs represent each of the 35 body segments. Each segment has three tabs representingcalculations in the transversal, coronal and sagittal planes except for thetail segments that only have a tab each for the transversal plane since tailbend was assumed not to contain a dorsoventral component (coronal plane) andhydrostatic balance in left/right axis (sagittal plane) is of less interestsince the specimen is not fixed in a posture with perfect bilateral symmetry.

**Additional file 10.** Physical demonstration of balance point. Thecoelacanth specimen was balanced in its preservation liquid (70% v/v ethanol)using lines anchored at the balance point. When the head is slightly lifted andthen dropped, the animal bounces back and forth until reaching its balancedequilibrium again. Frame rate is 30 frames/s.

**Additional file 11.** Interactive model of the segmented coelacanth specimen. To activate the 3D feature, click the model. Individual segments can be turn on/off and made transparent in the model three. Note that in order to maintain a left-to-right/snout-to-tail presentation of body measurements throughout text and figures, the imaging data has been horizontally flipped (mirrored). This has no effect on any calculations, but it means that what appears to be left pectoral and pelvic fins in the model are in fact right pectoral and pelvic fins and vice versa.

## Data Availability

CT and MRI data of the specimen *Latimeria chalumnae* (CCC 23) is publicly available on MorphoSource: https://www.morphosource.org/, media# 000398327, 000399052, 000399056, 000399068. Calculations on buoyancy and hydrostatic balance are available in Additional file 9.

## Abbreviations

adj.: Adjusted
ata: Atmospheres absolute
BMC: Bone mineral content
BMD: Bone mineral density
CCC: Coelacanth Conservation Council
COB: Center of buoyancy
COG: Center of gravity
CT: Computed X-ray tomography
EtOH: Ethanol
Form.: Formaldehyde
H. atl: *Hoploste* *thus* *atlanticus*
LC: Lipid content
L. cha: *Latimeria chalumnae*
MRI: Magnetic resonance imaging
N. fos: *Neoceratodus forsteri*
L. par: *Lepidosiren paradoxa*
P. ann: *Protopterus annectens*
*p*: Pressure
*s*: Salinity
TH: Total height
TL: Total length
unadj.: Unadjusted
uncat.: Uncategorized
