# Supplementary material for: Buoyancy and hydrostatic balance in a West Indian Ocean coelacanth Latimeria chalumnae
Source: BMC Biol. 2022 Aug 19;20:180. doi: 10.1186/s12915-022-01354-8 (PMC9389698; doi:10.1186/s12915-022-01354-8)
Supplement: Supplementary file 2 — Additional file 2. Additional results and discussion. [file 12915_2022_1354_MOESM2_ESM.pdf]

Additional results and discussion for

**Buoyancy and hydrostatic balance in a West Indian Ocean coelacanth *Latimeria chalumnae***

Henrik Lauridsen, Jens Mikkel Hyllested Pedersen, Steffen Ringgaard, Peter Rask Møller

## ADDITIONAL RESULTS AND DISCUSSION

### Bone mineral and lipid content measurements on preserved specimens

Before attempting to extract absolute measurements of bone mineral and lipid contents of the preserved coelacanth specimen using non-invasive MRI and CT imaging, we first needed to establish any potential effects that preservation may have on image acquisition as well as on actual bone mineral and lipid content. The coelacanth specimen under study was reportedly only preserved in ethanol, which was however unconventional at the time where an overwhelming majority of specimens were first fixed in 4% v/v formaldehyde via injections and then preserved in 70% v/v ethanol [43]. When comparing formaldehyde concentration in muscle tissue of the coelacanth specimen (*a*) with formaldehyde concentrations in muscle tissue of *Sparus aurata* specimens preserved in different ways, we found that the concentration in the coelacanth was not significantly different from *Sparus aurata* specimens fixed in 4% v/v formaldehyde and stored in 70% ethanol (*b*), but it was significantly higher than in both unfixed, frozen specimens (*c*) and ethanol preserved specimens stored together with formaldehyde fixed specimens (*d*) (i.e. potentially contaminated through the storage medium) (Additional file 3a) (One-way ANOVA: $F(3,6) = 39.36$, $p = 2.43 \times 10^{-4}$; Tukey HSD: *a* vs. *b*: $p = 0.70$, *a* vs. *c*: $p = 8.70 \times 10^{-4}$, *a* vs. *d*: $p = 0.017$, *b* vs. *c*: $p = 2.91 \times 10^{-4}$, *b* vs. *d*: $p = 0.012$, *c* vs. *d*: $p = 0.0104$). This leads us to conclude that the present coelacanth specimen was most likely fixed using formaldehyde in a similar fashion as most other

coelacanth specimens of the era. Since this method of protein crosslinking provides for a very stable specimen, this increases the usefulness of the aged coelacanth specimen in measurements of tissue components.

Buoyancy calculations in the coelacanth rely on quantification of bone mineral and lipid content in the specimen. Thus, the effect of storage in 70% v/v ethanol and imaging the specimen saturated with this storage solution was evaluated. Ethanol dilution series revealed an effect of ethanol concentration on acquired signal from both CT and MRI (Additional file 3b – 3c). X-ray attenuation of a substance is depended on density of the substance [20], and as the density of an ethanol/water mixture does not change linearly with an increase in ethanol concentration due to hydrogen bonding, a linear correlation was also not observed between x-ray attenuation (measured in Hounsfield units (HU) where attenuation of air is defined as -1000 HU and pure water as 0 HU) and ethanol concentration (Additional file 3b). In the ethanol concentration ([EtOH]) range of 20 – 100% v/v, a polynomic correlation described the relationship well ($HU = -0.0168 \times [EtOH]^2 - 0.4074 \times [EtOH] - 13.505$, $R^2 = 0.9978$) (Additional file 3b). Chemical analysis on a small tissue sample from the coelacanth specimen revealed a tissue concentration of ethanol of 37.38% v/v. Using the polynomic correlation from the dilution series, it was calculated that this tissue concentration results in an underestimation of signal of -52.2 HU due to the tissue water being replaced by less radiodense ethanol (Additional file 3b). The coelacanth storage medium containing 70% v/v ethanol scored a similar x-ray attenuation value as the control 70% v/v solution in the dilution series, indicating that the storage medium was not contaminated with other constituents that would change x-ray attenuation such as excessive amounts of dissolved lipids (Additional file 3b). Dixon MRI allows for the distinction between lipid and water in a sample or a specimen and thereby the calculation of lipid fraction on a voxel level. However, using a lipid free ethanol dilution series, we found the method sensitive to ethanol concentration (Additional

file 3c), and the relationship in the range of ethanol concentration from 0 – 90% v/v could be described well with an exponential correlation (Lipid fraction = $1.0806 \times e^{0.0396 \times [EtOH]}$, $R^2$ = 0.9765). Using this correlation, it was calculated that an ethanol concentration of 37.38% v/v as found in a tissue sample results in a 4.75% overestimation of lipid fraction (Additional file 3c).

Solubility of coelacanth lipids in 70% EtOH at different storage temperatures in unknown. In the lack of a sufficiently large coelacanth lipid sample, which would require unacceptable tissue destruction, we used pure oleyl oleate, the main wax ester found in coelacanth tissue [34], extracted swim bladder lipid of *Hoplostethus atlanticus*, which is dominated by wax esters quite similar to those of the coelacanth [41], and extracted whole body lipid of *Sparus aurata*, which is dominated by triglycerides and phospholipids [84], to measure solubility of specimen lipids in the storage solution (Additional file 3d). Solubility of all three lipids mixtures in 70% v/v ethanol was low, but increased with temperature in the 20 – 40 °C range that we deemed relevant to cover the range of reasonable storage temperatures (the higher end most likely only representing short-term storage temperatures in tropical climate without temperature control), and all three 70% v/v ethanol storage media were found to be close to saturated with lipids at room temperature (20 ˚C) (Additional file 3d). Still, even in a large 100 l storage container only 6.4 g oleyl oleate would dissolve in the store medium, thus most likely having a negligible effect on overall lipid content of the specimen.

Washout of significant amounts of bone mineral or lipids during 70% v/v ethanol storage was tested by comparing bone mineral and lipid content of *Sparus aurata* specimens preserved using either freezing (no chemical preservation) (*c*), 4% v/v formaldehyde fixation followed by 70% v/v ethanol storage (*b*) or direct preservation in 70% v/v ethanol (*d*). Although quantitative CT without ethanol correction underestimated bone mineral content following ethanol storage (*y*) (drop in red and blue curve in Additional file 3e), there was no significant

difference between initial bone mineral content ($x$) estimated using quantitative CT on the fresh specimens and the final bone mineral content ($z$) measured by ashing after 11 months in ethanol storage (Additional file 3e) (Two-way ANOVA with repeated measures for preservation effect, $F(2,12) = 19.44$, $p = 0.023$; Tukey HSD within treatment groups: $x_b$ vs. $y_b$: $p = 0.026$, $x_b$ vs. $z_b$: $p = 0.84$, $y_b$ vs. $z_b$: $p = 0.041$, $x_c$ vs. $y_c$: $p = 0.73$, $x_c$ vs. $z_c$: $p = 0.95$, $y_c$ vs. $z_c$: $p = 0.88$, $x_d$ vs. $y_d$: $p = 0.042$, $x_d$ vs. $z_d$: $p = 0.42$, $y_d$ vs. $z_d$: $p = 0.014$). Similarly, lipid content was not significantly affected by ethanol preservation (Additional file 3f) (One-way ANOVA: $F(2,6) = 0.33$, $p = 0.73$).

To validate that the water and lipid mapping using Dixon MRI on the ethanol preserved coelacanth specimen in fact yielded a correct mapping of water and lipid rich areas, respectively, we performed magnetic resonance spectroscopy (MRS) in slices across the entire specimen. The theoretically predicted and measured spectra of water and ethanol were found in water rich lean tissues like muscle and notochord, whereas the spectra of lipid rich tissues like post ocular tissue and in particularly the fatty organ were similar to the predicted spectrum of oleyl oleate (Additional file 4).
