# Supplementary figures and images for: Buoyancy and hydrostatic balance in a West Indian Ocean coelacanth Latimeria chalumnae
Source: BMC Biol. 2022 Aug 19;20:180. doi: 10.1186/s12915-022-01354-8 (PMC9389698; doi:10.1186/s12915-022-01354-8)

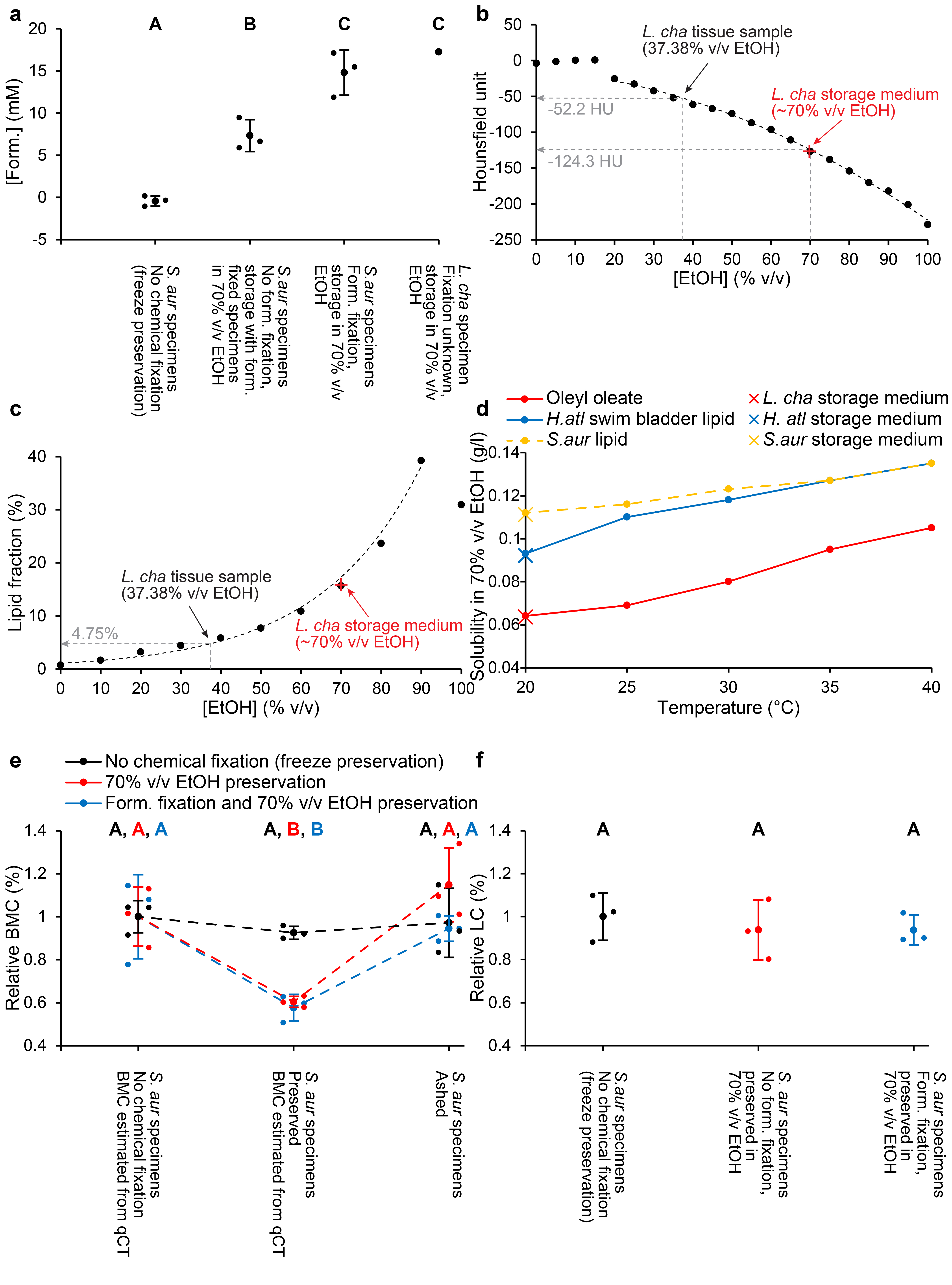

Supplement: Supplementary file 3 — Additional file 3. Chemical analyses on the effect of ethanolpreservation on actual bone mineral and lipid content and measurement errorusing CT and MRI. a, Formaldehyde(Form.) concentration in homogenized tissue samples of Sparus aurata (S. aur) indifferent states (fresh unfixed specimens, non-formaldehyde fixed but ethanol(EtOH) preserved specimens stored together with formaldehyde fixed specimen for11 months, and formaldehyde fixed and EtOH preserved specimens) compared to thetissue of the studied coelacanth (L. cha).The coelacanth in this study contains a similar amount of residual formaldehydein the tissue as formaldehyde fixed S.aur specimens. b, Hounsfieldunit of solutions with increasing concentrations of EtOH. c, Lipid fraction error from Dixon MRI of lipid free solutions withincreasing concentration of EtOH. A tissue concentration of EtOH of 37.38% v/vas measured in the coelacanth in this study results in a 4.75% error in thelipid fraction reading. d,Solubility in 70% v/v EtOH of oleyl oleate (most prevalent wax ester incoelacanth), swim bladder lipid of Hoplostethusatlanticus (H. atl) and full bodylipids of S. aur as a function oftemperature and lipid concentration in old storage medium. e, Relative bone mineral content (BMC) in S. aur specimens as estimated by quantitative x-ray computed tomography(qCT) before any fixation and after either direct EtOH preservation orformaldehyde fixation followed by EtOH preservation compared to measured BMCfrom ashing. Values are normalized to average BMC of each group in the freshunfixed state. Preservation in 70% v/v EtOH results in an underestimation(60.35%) of actual BMC due to the decreased x-ray attenuation in EtOH comparedto water (see b). Fixation andpreservation does not have significant effect on BMC of specimens (nosignificant difference for all groups between “No chemical fixation” and“Ashed”). Colored dashed lines are shown between categorical measurement pointsto highlight the paired nature of samples.f, R [file 12915_2022_1354_MOESM3_ESM.jpg]

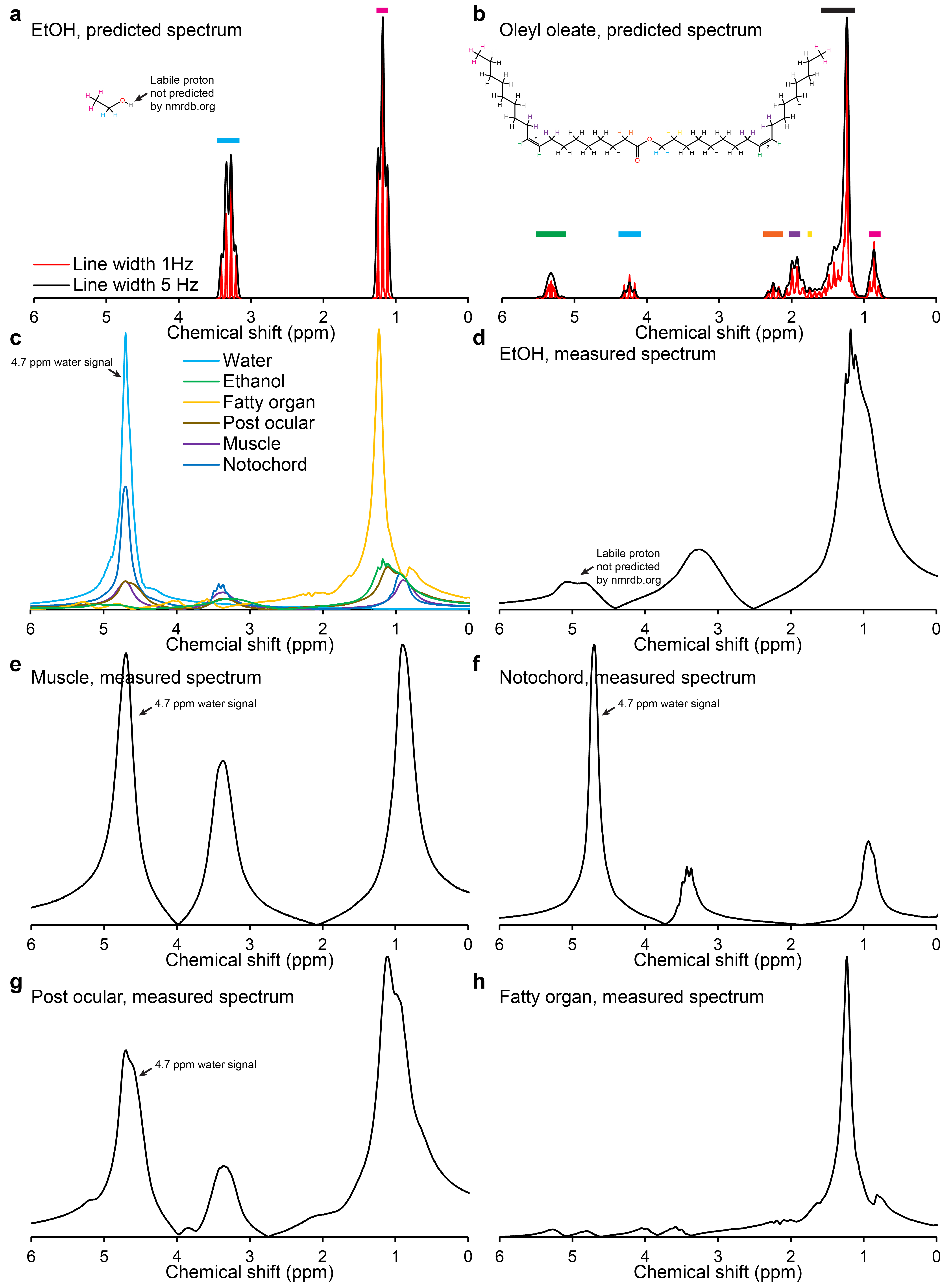

Supplement: Supplementary file 4 — Additional file 4. Magnetic resonance spectroscopy in the body ofthe coelacanth. a-b, Predictednuclear magnetic resonance spectra of ethanol (EtOH) (a) and the dominant wax ester, oleyl oleate (b), in the coelacanth. Spectra were prepared using both a linewidth of 1 Hz (red) to show individual peaks and 5 Hz (black) to betterrepresent results of low field strength spectra acquired in the specimen. c, Combined plot of acquired spectra inpure water, ethanol, fatty organ (caudal position, see Fig. 5a), post ocular,muscle, notochord. Since spectra were acquired with the same acquisitionparameters, the absolute values can be used as a proxy for proton density, i.e.,proton density of the 4.7 ppm water signal in pure water (light blue) is largeas expected and so is the proton density of methylene groups of wax esters inthe fatty organ (yellow). d-h,individual spectra of ethanol (d),muscle (e), notochord (f), post ocular (g), and fatty organ (h).While muscle and notochord are dominated by water and ethanol signals, postocular tissue and especially the fatty organ is more dominated by the lipid signal. [file 12915_2022_1354_MOESM4_ESM.jpg]

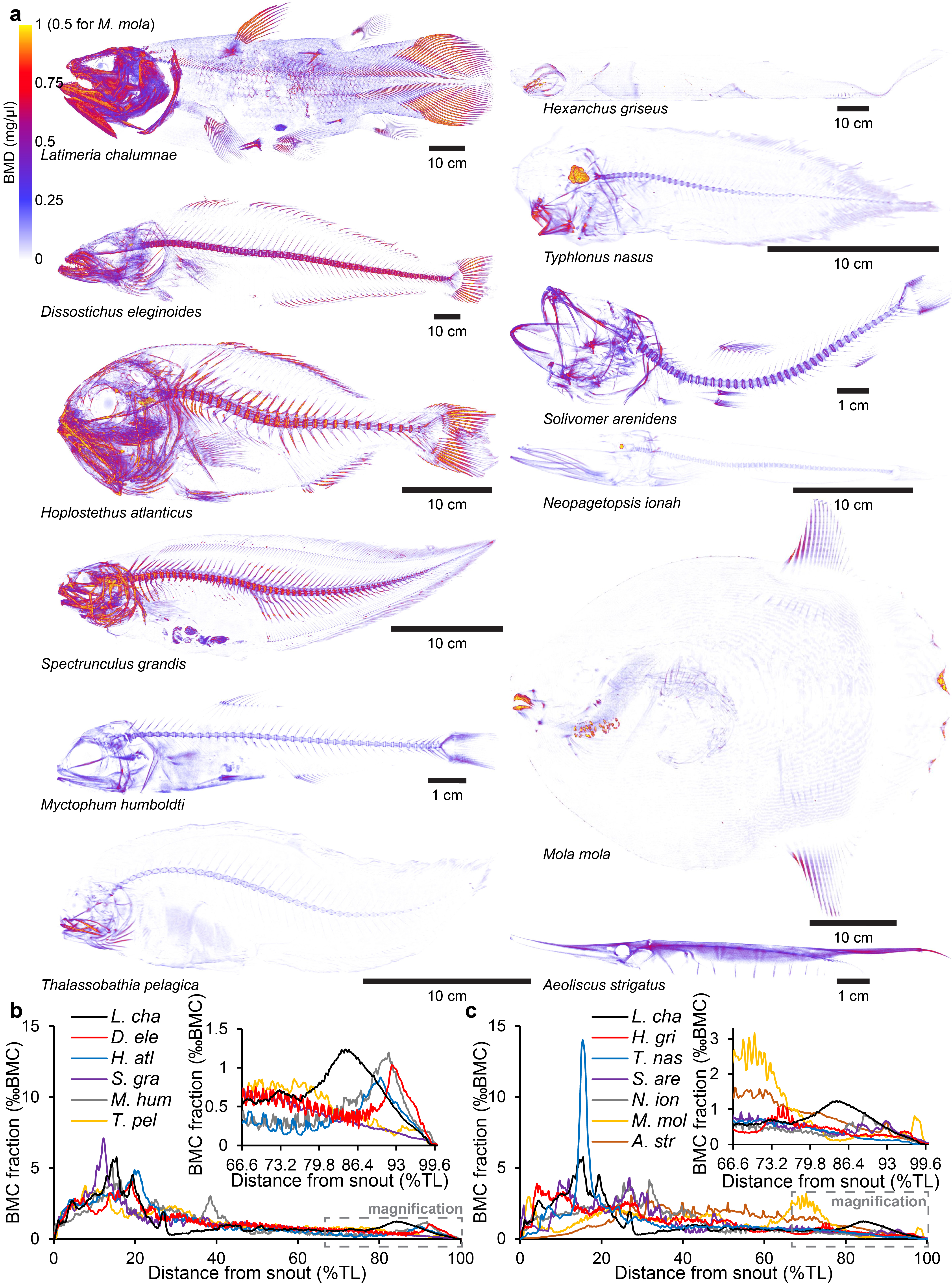

Supplement: Supplementary file 5 — Additional file 5. Distribution of bone mineral in some species ofosteichthyes and chondrichthyes with reported neutral or near neutral buoyancyor pelagic or deep-sea lifestyles that would suggest so. a, Volume rending of bone mineral density (BMD) map of thecoelacanth, ten species of osteichthyes and one species of chondrichthyes.Species are ranked according to the similarity of bone mineral distributionrelative to the coelacanth (Dissostichuseleginoides most and Aeoliscusstrigatus least similar). Dissostichuseleginoides, Neopagetopsis ionah,Mola mola, Aeoliscus strigatus and Hexanchusgriseus are reportedly neutrally or close to neutrally buoyant [21, 86–88]. Hoplostethus atlanticus, Myctophum humboldti and Solivomer arenidens contain a lipidfilled swim bladder proposed to have a similar function as the fatty organ ofthe coelacanth [58], whereas Dissostichuseleginoides, Neopagetopsis ionah,Mola mola and Hexanchus griseus don’t contain any swim bladder. Spectrunculus grandis, Thalassobathia pelagica and Typhlonus nasus may conduct similar headdown hunting maneuvers similar to what is observed in the coelacanth. Note thatBMD color bar for Mola mola spans 0 –0.5 mg/µl. b, Distribution of bonemineral content (BMC) fraction of total BMC along the total length (TL) in thespecies above the panel (the black graph for the coelacanth, L. cha, is present in both panels forcomparison). Dashed gray squares in the most caudal 33% portion of the graphsare magnified in the insertions to highlight species with similarly mineralizedcaudal fins as the coelacanth. [file 12915_2022_1354_MOESM5_ESM.jpg]

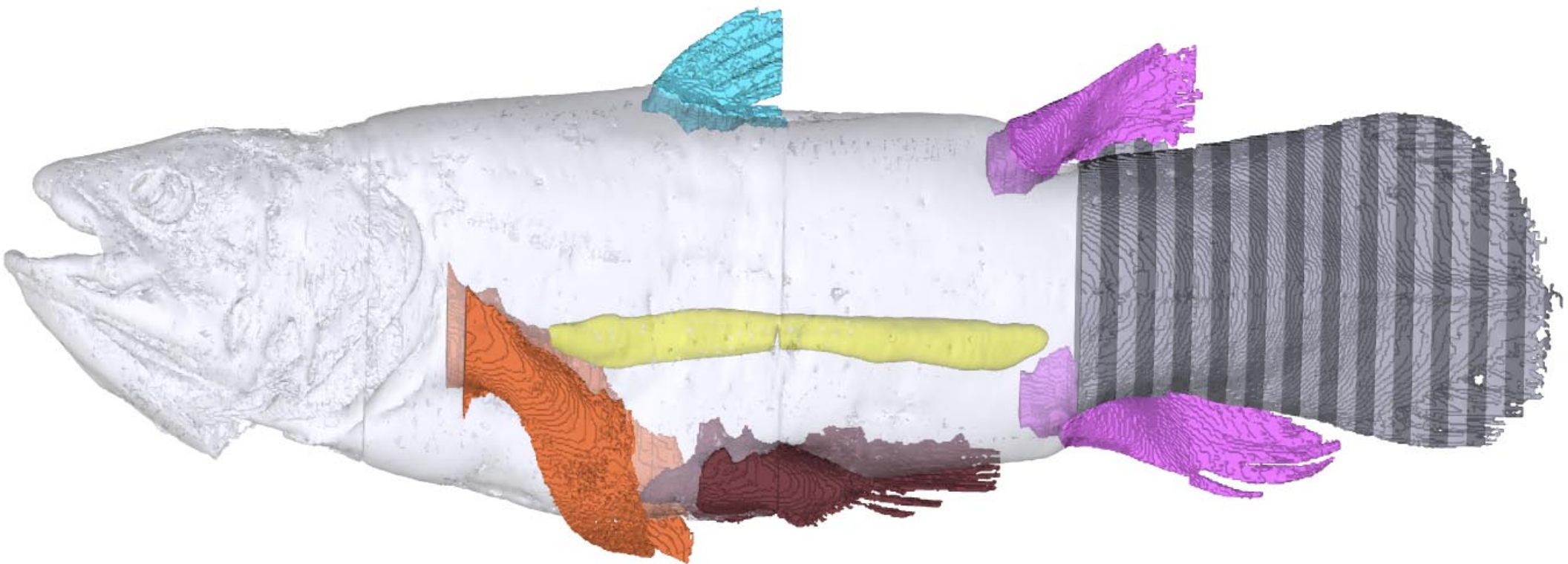

Supplement: Supplementary file 11 — Additional file 11. Interactive model of the segmented coelacanth specimen. To activate the 3D feature, click the model. Individual segments can be turn on/off and made transparent in the model three. Note that in order to maintain a left-to-right/snout-to-tail presentation of body measurements throughout text and figures, the imaging data has been horizontally flipped (mirrored). This has no effect on any calculations, but it means that what appears to be left pectoral and pelvic fins in the model are in fact right pectoral and pelvic fins and vice versa. [file 12915_2022_1354_MOESM11_ESM.pdf]
